# Fires prime terrestrial organic carbon for riverine export to the global oceans

**DOI:** 10.1038/s41467-020-16576-z

**Published:** 2020-06-03

**Authors:** Matthew W. Jones, Alysha I. Coppola, Cristina Santín, Thorsten Dittmar, Rudolf Jaffé, Stefan H. Doerr, Timothy A. Quine

**Affiliations:** 1 Tyndall Centre for Climate Change Research, School of Environmental Sciences, University of East Anglia, Norwich, UK; 2 Department of Geography, University of Zurich, Zürich, Switzerland; 3 Geological Institute, Department of Earth Sciences, ETH Zürich, Sonneggstrasse 5, 8092, Zürich, Switzerland; 4 Geography Department, College of Science, Swansea University, Swansea, UK; 5 Biosciences Department, College of Science, Swansea University, Swansea, UK; 6 Research Group for Marine Geochemistry (ICBM-MPI Bridging Group), Institute for Chemistry and Biology of the Marine Environment (ICBM), University of Oldenburg, Oldenburg, Germany; 7 Helmholtz Institute for Functional Marine Biodiversity (HIFMB), University of Oldenburg, Oldenburg, Germany; 8 Southeast Environmental Research Center and Department of Chemistry and Biochemistry, Florida International University, Miami, FL USA; 9 Geography Department, College of Life and Environmental Science, University of Exeter, Exeter, UK

**Keywords:** Carbon cycle, Carbon cycle, Geochemistry, Marine chemistry

## Abstract

Black carbon (BC) is a recalcitrant form of organic carbon (OC) produced by landscape fires. BC is an important component of the global carbon cycle because, compared to unburned biogenic OC, it is selectively conserved in terrestrial and oceanic pools. Here we show that the dissolved BC (DBC) content of dissolved OC (DOC) is twice greater in major (sub)tropical and high-latitude rivers than in major temperate rivers, with further significant differences between biomes. We estimate that rivers export 18 ± 4 Tg DBC year^−1^ globally and that, including particulate BC fluxes, total riverine export amounts to 43 ± 15 Tg BC year^−1^ (12 ± 5% of the OC flux). While rivers export ~1% of the OC sequestered by terrestrial vegetation, our estimates suggest that 34 ± 26% of the BC produced by landscape fires has an oceanic fate. Biogeochemical models require modification to account for the unique dynamics of BC and to predict the response of recalcitrant OC export to changing environmental conditions.

## Abstract (web summary)

Black carbon is a recalcitrant and unique form of organic carbon formed from incomplete combustion. Here the authors use global sampling to reduce uncertainty in the flux of terrestrial black carbon to the oceans, predicting that 34% of black carbon produced by fires has an oceanic fate.

## Introduction

Globally, terrestrial net primary production (NPP) sequesters around 60 Pg C year^−1^ from the atmosphere to stocks of organic carbon (OC) in biomass^1–3^. The majority of this carbon returns to the atmosphere from the terrestrial biosphere over decadal timescales, through fire emissions, herbivory or by entering soils as dead organic matter and undergoing microbial decomposition^4–6^. Only around 1% of terrestrial NPP is exported to the global oceans by rivers (300–800 Tg C year^−1^)^7–9^. Some fractions of the exported OC are bio-labile and thus drive coastal and marine food webs, while other major fractions are biologically recalcitrant and have potential for long-term storage in oceanic pools^10–15^.

Black carbon (BC) is a quantitatively significant by-product of incomplete combustion of terrestrial OC. Around 40–215 Tg BC year^−1^ are generated by the incomplete combustion of biomass during landscape fires, forming a major component of residual charcoal and ash deposits (see “Methods”)^16^. An additional 2–29 Tg BC year^−1^ are emitted as soot (aerosol) from landscape fires and fossil fuel combustion^17,18^. The BC produced by landscape fires has three potential fates. First, it can be mineralised through biotic or abiotic processes^19,20^, such as microbial decomposition or combustion during subsequent fires^21,22^. Second, it can reach deep soil stores where its decomposition is exceptionally limited^23,24^. Third, it can be exported to the global oceans as a fraction of riverine dissolved organic carbon (DOC) or particulate organic carbon (POC)^25,26^.

**Fig. 1:**
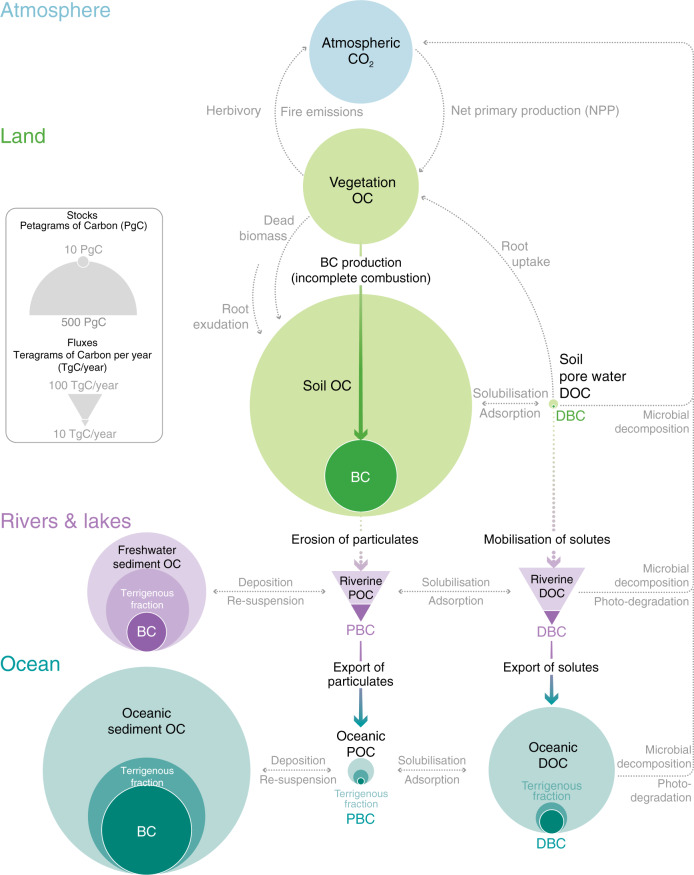
Global dynamics of black carbon (BC) and organic carbon (OC). DOC dissolved organic carbon, DBC dissolved black carbon, POC particulate organic carbon, PBC particulate black carbon. The relative size of the stocks and fluxes of OC and BC in soils, sediments, dissolved organic matter and suspended particulates are indicated by the size of the circles (stocks) and triangles (fluxes). BC is selectively preserved across the land-to-ocean aquatic continuum and thus represents a large fraction of the terrigenous OC in ocean pools. Rivers are the primary source of oceanic BC, though small additional fluxes of aerosol BC (soot) to the ocean surface are not shown. The estimates for each stock and flux are provided in Supplementary Dataset along with references to source data.

BC is among the most recalcitrant forms of OC in the Earth System^27,28^. Its poly-condensed aromatic molecular structure renders it relatively inaccessible to microbial organisms^19,20,29^. Owing to its biological recalcitrance, BC accumulates in Earth’s major OC pools^27,28,30–32^. Despite the annual production of BC equating to <0.5% of OC production by the terrestrial biosphere (~60 Pg C year^−1^)^1–3^ and marine organisms (~13 Pg C year^−1^)^2^, BC constitutes ~5–18% of global soil OC stocks^33^, ~2–5% of oceanic DOC stocks^34,35^ and ~5–30% of the OC in ocean sediments^27,36,37^ (Fig. 1). Estimates of the BC contribution to global oceanic DOC stocks do not differ substantially from estimates of the total terrigenous fraction of DOC (<5%)^14^. Similarly, estimates of the BC contribution to the global stock of OC in oceanic sediments do not differ substantially from estimates of the terrigenous fraction of the total OC in oceanic seciments (approximately one-third)^36,38^. Hence, a considerable fraction of the terrigenous OC stored in the global oceans is thought to have been altered by fire (Fig. 1). The residence time of oceanic BC stocks is on the order of millennia to tens of millennia^30,35,39,40^, likely an order of magnitude longer than soil BC stocks (centuries to millennia)^19,20^ and multiple orders of magnitude longer than terrestrial OC (decades to centuries)^4–6^. Hence, the production of BC and its subsequent export to the global oceans significantly extends the residence time of terrigenous carbon in the Earth System.

Riverine dissolved black carbon (DBC) is chiefly a by-product of the decomposition of soil BC stocks, which are maintained by landscape fires and by small aerosol fluxes to the land surface^41,42^ (Fig. 1). Like OC more generally, the rate at which soil BC degrades is moderated by environmental factors, such as temperature, hydrology, soil characteristics (e.g. clay content, mineralogy and pH) and land use^33,42–45^. Nonetheless, the disproportionate effect of these factors on rates of soil BC decomposition relative to bulk soil OC decomposition has been shown to drive variation in the DBC content of riverine DOC at regional scales^42,45^. In addition, BC production is more concentrated in the tropics than the production of biogenic OC through NPP. While ~50–60% of terrestrial NPP occurs in tropical forests and savannahs^1^, ~80% of global BC production occurs in these biomes^16^. These factors indicate that the catchment dynamics of BC differ from those of unburned OC and promote the expectation of global-scale variability in the DBC content of riverine DOC. Nonetheless, Jaffé et al.^26^ previously identified a simple linear relationship between the concentrations of DBC and DOC in global rivers, which suggested that DBC constitutes ~10% of the global riverine flux of DOC. Deviations from the global average DBC content of DOC were observed in individual river catchments; however; deviance from the 10% average was low across the global sample of 109 observations (±1% in absolute terms, incorporating standard error of the regression coefficient errors)^26^.

Here, using an extended global data set of 409 DBC and DOC concentration measurements (Supplementary Dataset)^26,42,43,45–56^, we evaluate global-scale variability in the DBC content of riverine DOC across latitudes and biome boundaries and extrapolate these spatial patterns to estimate global riverine DBC export. Our data set incorporates 409 coupled measurements of DOC and DBC concentration in total, including 195 from 34 major rivers and 214 from 44 minor channels (Supplementary Table 1). The data set incorporates 300 new coupled measurements from 12 major rivers and 90 minor channels. Four hundred and five of the data points represent individual samples taken at a single time, while four of the data points represent the average of samples collected over multiple seasons. The data set includes 204 new samples from (sub)tropical rivers (<30° N/S), which is an important advance because ~90% of global BC production occurs in the (sub)tropics^16^ and because (sub)tropical rivers contribute ~60% of the total global DOC export flux (128 ± 20 Tg C year^−1^ of 205 ± 21 Tg C year^−1^)^8^. We combine our estimate for global DBC export with an existing estimate for particulate BC (PBC) export^25^ to calculate the total global export of BC by rivers.

## Results and Discussion

### BC content of riverine DOC

**Fig. 2:**
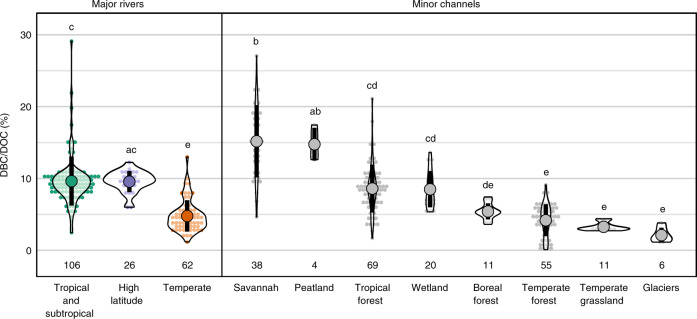
Dissolved black carbon (DBC) content of dissolved organic carbon (DOC; %). Each observational value (see Supplementary Dataset) is marked as a small dot and classified by channel type. These observations are stacked horizontally as in a histogram with a grouping interval of 0.5%, while the violin plot marks the kernel probability density at the range of observed values. The mean and standard deviation of observed values are marked by a large dot and thick black line, respectively. The number of data points included in each channel class is also shown. Letters denote groups with statistically similar mean values according to Tukey honest significant difference (HSD) test.

Our data set reveals significant and structured global-scale variability in the DBC content of DOC, with tropical and high-latitude rivers exporting DBC in greater ratios than temperate ones. Significant latitudinal differences were observed between major rivers in different latitude ranges (Fig. 2; Supplementary Table 1). The DBC content of DOC was, on average, twice greater in major (sub)tropical (9.7 ± 1.5%) and high-latitude rivers (9.6 ± 1.5%) than in major temperate rivers (4.6 ± 2.0%). The DBC content of DOC was also significantly greater in minor channels draining savannah (15.2 ± 5.0%) and peatland (14.8 ± 2.3%) than in channels draining tropical forest (8.6 ± 3.3%) and wetland (8.3 ± 2.7%) and in all of these channel classes when compared to channels draining temperate forest (4.4 ± 2.5%), temperate grassland (3.3 ± 0.6%) and glaciers (2.1 ± 1.0%). The DBC content of DOC in rivers draining boreal forest (5.4 ± 1.1%) was neither significantly lower than in channels draining tropical forest and wetland nor significantly greater than in channels draining temperate forest, temperate grassland and glaciers. These systematic differences in the DBC content of DOC demonstrate that the dynamics of BC and OC are not uniformly coupled across biomes and latitudes.

Differences in the average DBC content of DOC are greater between minor channels spanning different biomes than in major rivers spanning latitude bands (Fig. 2). Jaffé et al.^26^ previously suggested that variability in the DBC content of DOC in minor channels attenuates when integrating over large catchments, and in support, our data set reveals some instances in which variation in the DBC content of DOC in major rivers can be explained by variability in the biome composition of their catchments. For example, we identified a linear relationship between peatland extent and the DBC content of DOC in the 6 major high-latitude rivers included in our global data set (*R*^2^ = 0.68, *p* < 0.05; Supplementary Fig. 1)^57^. We suggest that this relationship occurs because the DOC in channels draining peatlands is enriched in DBC (14.8 ± 2.3%) relative to channels draining boreal forest (5.4 ± 1.1%) and glaciers (2.1 ± 1.0%) (Fig. 2).

### Global riverine export of BC

Our estimate for the global export of DBC by rivers is 18.0 ± 3.9 Tg C year^−1^. This estimate is lower than that made previously by Jaffé et al.^26^ (26.5 ± 1.7 Tg C year^−1^), which is principally because we used lower estimates for the global riverine flux of DOC that derive from a more recent meta-analysis of river discharge and DOC concentration data^8^. Although our central estimate also suggests that DBC constitutes a smaller fraction of the global riverine DOC flux (8.8 ± 2.1% of 205 ± 21 Tg DOC year^−1^)^8^ than that of Jaffé et al.^26^ (10.6 ± 0.7% of 250 Tg DOC year^−1^)^38^, this difference was not statistically significant.

**Fig. 3:**
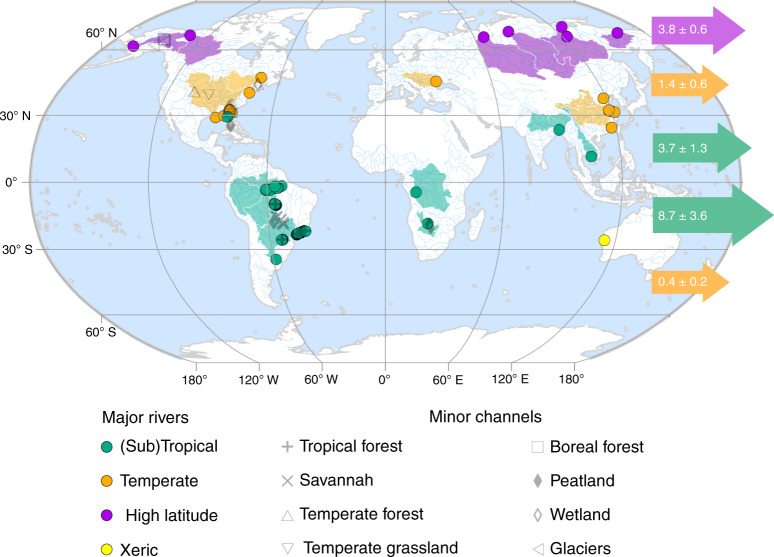
Global map of the sampling locations represented by our global data set. Dissolved organic carbon (DOC) and dissolved black carbon (DBC) concentrations are available for each location (Supplementary Dataset). Sampling locations within major river systems, whose catchment areas exceed 10,000 km^2^ (see “Methods”) are classified as (sub)tropical, temperate or high latitude according to the Freshwater Ecosystems of the World data set^88^. The catchment areas of these major rivers are also shown. One major xeric river system is represented in the data set but not used in the calculation of global DBC export. Minor channels draining specific biomes are classified as described in the primary literature. Horizontal arrows indicate the global flux of DBC from each 30° latitude range (Tg C year^−1^).

Nonetheless, recognising that the DBC content of riverine DOC varies globally leads to a marked difference in the latitudinal distribution of DBC export. Using latitude-specific estimates for DOC export from Dai et al.^8^ and the distribution of the DBC content of DOC in major rivers (Fig. 2), we estimate that (sub)tropical rivers export 69 ± 26% of the total global riverine flux of DBC (12.4 ± 3.8 Tg C year^−1^), while northern high-latitude rivers export 21 ± 6% (3.8 ± 0.6 Tg C year^−1^) and temperate-latitude rivers just 10 ± 4% (1.8 ± 0.6 Tg C year^−1^; Fig. 3; Supplementary Table 2). The large flux of DBC from (sub)tropical latitudes relates both to the large DOC export flux in this region (62 ± 12% of the global DOC flux) and the relatively high contribution of DBC to riverine DOC exhibited by the rivers in this latitude range (Fig. 2; Supplementary Table 1). We find that the use of a single global relationship between DBC and DOC for the prediction of DBC export would result in a 54–75% (1.0–1.3 Tg C year^−1^) overestimation of DBC export by temperate rivers, a 16–22% (2.0–2.8 Tg C year^−1^) underestimation of export by (sub)tropical rivers and a 10–15% underestimation of export by high-latitude rivers (Supplementary Table 2).

Our estimates account for the organic products of fire whose recalcitrance is considered to differ substantially from that of unburned vegetation (see “Methods”). We do not account for the export of other soluble products of low-temperature vegetation charring, such as levoglucosan, which have short residence times compared to those of the DBC and thus lesser significance for the oceanic storage of terrigenous carbon^58–60^.

The data set on which our global export estimates are based captures global-scale spatial variability in the DBC content of riverine DOC. Our estimates do not explicitly consider the influence of hydrology and its seasonal variation on the DBC content of riverine DOC. The DBC content of riverine DOC was previously shown to vary across hydrological gradients in one river system; specifically, the Yellow River, where DBC content of DOC varied between 1% in winter and 13% in the high spring flow of 2015^52^. Nonetheless, most studies have failed to identify significant seasonality in the DBC content of DOC, including in major high-latitude rivers^48^, tropical rivers and temperate catchments^51,56^. Soil moisture, a proxy for seasonal hydrology, was also not found to be a significant driver of variability in the DBC content of DOC in tropical Brazilian rivers^42^. We verified that the differences in the DBC content of DOC across latitudes and biomes are not due to bias in the timing of sample collection. Such a bias could, in theory, influence the spatial variability that we observe across river classes if one river class disproportionately includes samples from periods with a high DBC content of DOC relative to other river classes. However, we found no evidence for significant variability in the DBC content of DOC across seasons within river classes collated at the global scale (Supplementary Fig. 2) and hence conclude that seasonality in the DBC content of DOC was insufficient to significantly bias the spatial differences between river classes that we observed in our data set.

By combining our global estimate for riverine DBC export with an existing estimate riverine PBC export ($25_{ - 9}^{ + 14}$ Tg C year^−1^)^25^, we estimate that rivers export a total of 43 ± 15 Tg BC year^−1^ to the global oceans. This is equivalent to 12 ± 5% of total riverine OC export to the global oceans (362 ± 77 Tg C year^−1^; see “Methods”)^8,9^. While the riverine export of OC amounts to just 0.6 ± 0.1% of the biogenic OC sequestered annually by terrestrial NPP (60 Pg C year^−1^), the riverine export of BC amounts to 34 ± 26% of the BC produced annually by landscape fires (128 ± 84 Tg C year^−1^; see “Methods”)^16,27^. This comparison demonstrates that a considerably greater fraction of the BC produced by landscape fires has an oceanic fate than the unburned OC sequestered by terrestrial NPP.

The uncertainty range of our DBC export estimate represents our best estimate of 1*σ* uncertainty (i.e. we consider there to be a 68% likelihood of the export fluxes falling within the stated bounds; see “Methods”). The 1*σ* uncertainty of the DBC content of DOC is 35% in (sub)tropical samples, 43% in the temperate samples and 15% in high-latitude samples (Fig. 2). Meanwhile, the 1*σ* uncertainty of global DOC export fluxes is 16% for (sub)tropical rivers, 9% for temperate rivers and 8% for high-latitude samples (Supplementary Table 3). The moderately high uncertainty in the DBC content of DOC and the DOC export flux in the (sub)tropics, combined with the large contribution of tropical rivers to global DOC export, means that most of uncertainty in the global DBC export flux derives from the (sub)tropical flux. For our global estimate of total BC export, most of the global uncertainty derives from the existing estimate of PBC export, whose 1*σ* uncertainty exceeds 50%.

Recently, oceanic DBC was found to be ~6‰ enriched in δ^13^C relative to riverine DBC, possibly indicating that there are significant non-pyrogenic sources of DBC to the global oceans^61^. Hypothesised DBC sources may include: a biogenic source in ocean waters^61^, a petrogenic source at the ocean bed or in coastal sediments (e.g. asphalt or aquifer brine)^40^, and thermogenic matter from hydrothermal vents^62,63^. We add that BC produced by the burning of plants with a C_4_-type photosynthetic pathway would also be depleted in δ^13^C and that it may enter the ocean DBC pool either via solubilisation of soil BC or exported PBC^64^ or by direct aerosol deposition to the ocean surface^65,66^. As around 50% of pyrogenic carbon is produced in savannah environments^16^ and rivers draining savannahs are especially enriched in DBC (Fig. 2), the latter scenario does not seem unlikely. Moreover, it remains plausible that the isotopic fractionation occurs during the partial photo-degradation of DBC from terrigenous, pyrogenic sources^61^. Photo-degradation is known to cause a 1–4.5‰ enrichment of freshwater DOC in δ^13^C^67,68^ and DBC is considered to be particularly susceptible to photo-degradation^69^; however, the effect of photo-degradation on the δ^13^C of DBC is yet to be quantified. Future work should seek to discount these plausible sources of δ^13^C-depleted pyrogenic DBC to the global oceans.

### Environmental change and BC export

Over the past 20 years, a 24% net reduction in global burned area has been driven chiefly by the conversion of savannahs to agricultural land^70–72^. Nonetheless, burned area has increased in forested regions with high BC production rates per unit area, and thus global rates of BC production by landscape fires showed no trend in the past two decades^16^. According to fire models, global burned area is likely to increase in the coming centuries, with this increase concentrated in forests and in regions where the human capacity to suppress fire fails to keep pace with increasing ignition frequency^73–76^. Through their control on the rates of BC input to soils and waters, these historical and future changes in global fire incidence may have cascading impacts on terrestrial BC stocks, the riverine export of BC and the placement of BC into oceanic storage.

Observations of riverine and oceanic BC have accumulated over the past two decades and represent a narrow snapshot in time relative to the residence time of BC in the Earth System. It is thus not feasible to detect trends in contribution of BC to the total fluxes of OC exported by rivers with the available data. Until a longer timeline of observational data becomes available, it will only be possible to quantify historical and future changes to BC export using models that reliably reproduce the spatial patterns of BC and OC export observed in the available snapshot. Process-based models are already used to investigate the effects of global climate and land use changes on the catchment dynamics of OC, including its riverine export^77–79^, yet these have been under-utilised for the study of BC dynamics. Modelling of the processes leading to DBC export is especially needed because these processes disconnect the dynamics of BC from the physical mobility of sediments and are instead controlled by a range of interacting hydrological and biogeochemical factors that affect the solubilisation and lateral transfer of organic matter (Fig. 1)^42,59,77,78^.

Until now, there has been little incentive to adapt process-based models to explicitly represent the riverine export of DBC because the DBC content of DOC has been considered constant with respect to fire regime and climate^26,59^. If the DBC content of riverine DOC is consistent across spatial gradients of fire, climate and other environmental factors, then why should this be expected to respond to temporal changes in such factors? One major finding of our work is that the DBC content of DOC is spatially variable across environmental gradients, with the further implication that these variations are important to understanding historical and future changes in the DBC content of DOC. Our results highlight the pressing need to construct process-based models that explicitly account for the unique biogeochemical properties and dynamics of BC in river catchments and in the global oceans. This will enable prediction of the impact of changes in climate and fire regime on the source to sink dynamics of BC and the implications for net carbon exchange between the Earth’s surface and the atmosphere.

## Methods

### BC content of riverine DOC

The global data set of DBC and DOC measurements analysed here was extended from that of Jaffé et al.^26^, which included 109 data points from published^43,49,50,80–82^ and unpublished sources. We added 300 new data points, including from Coppola et al.^54^ (12 measurements), Jones et al.^42^ (78 measurements), Bao et al.^55^ (18 measurements), Roebuck et al.^45^ (19 measurements), Roebuck et al.^56^ (13 measurements), Marques et al.^53^ (114 measurements), Wang et al.^52^ (15 measurements), Wagner et al.^51^ (30 measurements) and Mannino and Harvey^46^ (1 measurement).

The DBC concentration of all samples was measured using either the benzene polycarboxylic acid (BPCA) approach^83^ or the chemo-thermal oxidation (CTO)^84^ approach (specific details are provided for each measurement in the Supplementary Dataset). These approaches are each widely employed in the measurement of BC in environmental samples from soils, sediments and aquatic solutions^37,59,84,85^. The BPCA approach detects a spectrum of aromatic moieties with varying degrees of poly-condensation, which are considered to be produced by the combustion of a wide range of fuels (e.g. woody and non-woody biomass, as well as fossil fuels) at a wide range of temperatures (~300–>1000 °C)^84,86,87^. Meanwhile, the CTO approach detects the most poly-condensed BC structures, which are typically associated with the BC produced in the form of soot or charcoal produced at particularly high temperatures^37,84^. Inter-comparisons of BC concentration measurements deriving from these techniques, among others, have been completed elsewhere (refs. ^84,85^). The data set collated for the purpose of the current study is dominated by measurements obtained using the BPCA approach (393 measurements). Sixteen measurements were obtained by applying the CTO approach to samples from three major temperate rivers across two studies^46,52^. No significant difference was observed between the DBC content of DOC in these samples (4.6 ± 3.2%) and the 46 measurements obtained by 6 studies that applied the BPCA approach to samples from 7 other major temperate rivers (4.9 ± 1.7%).

As both the BPCA and CTO techniques quantify poly-condensed aromatic forms of ‘pyrogenic’ carbon in charcoal, soot and ash, we consider both to be valid methods for the quantification of the biologically recalcitrant BC that exhibits exceptional storage times in the aquatic pools. Therefore, we include measurements made using both approaches in our data set and suggest that inter-method variability in DBC measurements should be treated as one source of the uncertainty present in our statistical analysis of data distributions. On the other hand, we exclude one study that measured levoglucosan concentrations in riverine DOC^58^ on the basis that levoglucosan is a marker of low-temperature biomass charring and is bio-labile^60^; in contrast to poly-condensed aromatic carbon, this portion of the pyrogenic carbon continuum has low potential for long-term storage in aquatic systems^59^. While other approaches to quantifying poly-condensed aromatic carbon concentrations are available^84,85^, these are yet to be used to measure riverine DBC concentrations.

Measurements of DBC and DOC in samples from major rivers, whose catchment areas exceed 10,000 km^2^, were assigned to classes of the Freshwater Ecoregions of the World scheme^88^: polar freshwaters, temperate lowland, temperate upland & lowland, (sub)tropical lowland, (sub)tropical upland, (sub)tropical upland & lowland, xeric. The multiple temperate and (sub)tropical classes were grouped for the estimation of DBC export. For minor channels, the biome in which the study catchment occurred was adopted based on descriptions given in the primary studies or by cross-referencing sampling locations with a map of the Terrestrial Ecosystems of the World^89^.

### OC export

The latitudinal DOC export fluxes used in our calculation were as reported by Dai et al.^8^ (205 ± 21 Tg C year^−1^, including 128 ± 20 Tg C year^−1^ from tropical rivers, 38 ± 4 Tg C year^−1^ from temperate rivers and 39 ± 3 Tg C year^−1^ from the high latitudes) and are based on global-scale extrapolation of DOC concentrations (mg L^−1^) in 118 world rivers and long-term average river discharge data from the world’s largest 925 exorheic rivers.

For the calculation of total OC export fluxes, we summed existing global estimates of DOC export from Dai et al.^8^ (205 ± 21 Tg C year^−1^) and POC export from Galy et al.^9^ ($157_{ - 50}^{ + 74}$ Tg C year^−1^). For the calculation of uncertainty in the total OC export fluxes, we converted the nonparametric uncertainty in the POC export flux to a synthetic normal distribution bounded by the largest difference between the median and the upper and lower quartiles (±74 Tg C year^−1^) and subsequently added the DOC and POC flux uncertainties in quadrature. We present the uncertainty in the total OC flux as a 1*σ* uncertainty range (i.e. there is a 68% probability of the value falling within the stated range) but note that the lower bound is conservative. For the expression of the total OC export flux as a fraction of the global terrestrial OC production flux by NPP, our estimate of the total OC flux was divided by global terrestrial NPP (60 Pg C year^−1^; after Huston and Wolverton^1^), and uncertainties were computed in quadrature.

### DBC export

Our estimate for global DBC export is based on the application of the average (±standard deviation) DBC content of DOC observed in major (sub)tropical, temperate and high-latitude rivers to the latitude-specific estimates of DOC export (ref. ^8^; Supplementary Note 1). We opted for this approach, over the option of fitting a global relationship between DBC and DOC concentrations, because we identified and avoided procedural challenges to the fitting of a simple linear regression model to this data set (Supplementary Note 1). Specifically, in order to force the residual errors of the fitted simple linear model conform to the assumption of heteroscedasticity, a measurement binning approach is required in which samples with similar DOC concentrations are grouped and the mean DOC and DBC concentration values of these groups are used as data points for statistical model fitting (as in ref. ^26^). This binning approach masks a portion of the variance present in the global data set of DOC and DBC concentrations and grants undue influence to samples from high-latitude rivers (Supplementary Note 1). To evaluate the sensitivity of DBC export estimates to method of calculation, we compare our estimate with that resulting from a different global DOC flux estimate and the model-fitting approach of Jaffé et al.^26^ (Supplementary Note 2; Supplementary Tables 3–7; Supplementary Fig. 3).

We used only the observations of DBC content of riverine DOC in major rivers in our calculations of global DBC export, as opposed to all measurements of the DBC content of riverine DBC in all samples. An alternative approach would be to consider the biome composition of river catchments globally and to use this as a weighting for the central and uncertainty statistics representing the DBC content of riverine DOC in the rivers draining those biomes. We chose not to adopt the alternative approach on the basis that the distribution of sampling regions within biomes was generally restricted to small areas of the global extent of those biomes. For example, the studies of minor channels draining tropical forests and savannahs are restricted to the continent of South America (Fig. 3). In contrast, measurements from the major rivers have a global coverage in all latitude bands (Fig. 3), and thus we consider these suitable for global-scale extrapolation.

To calculate uncertainty in latitudinal DBC export fluxes, uncertainties in DOC export fluxes and the DBC content of DOC are multiplied in quadrature and represent a 1*σ* uncertainty estimate. The uncertainty in global DBC export fluxes is calculated by summing the 1*σ* uncertainties in DBC export from tropical, temperate and high latitudes in quadrature.

### Total BC export

For calculation of total BC export fluxes, we summed our global estimate of DBC export and the previously published estimate of PBC export of Coppola et al.^25^ ($25_{ - 9}^{ + 14}$ Tg C year^−1^). The uncertainty range of the PBC export estimate was increased slightly to account for variability in the PBC content of POC reported by Coppola et al.^25^ in addition to the uncertainty in the global POC export flux as presented by Galy et al.^9^ ($157_{ - 50}^{ + 74}$ Tg C year^−1^). For the calculation of uncertainty in the total OC export fluxes, we converted the nonparametric uncertainty in the PBC export flux to a synthetic normal distribution bounded by the largest difference between the median and the upper and lower quartiles (±14 Tg C year^−1^), subsequently added the DBC and PBC flux uncertainties in quadrature. We present the uncertainty in the total BC export flux as a 1*σ* uncertainty range, but note that the lower bound is conservative. For the expression of the total BC export flux as a fraction of the global terrestrial BC production by landscape fires, our estimate for the total BC export flux was divided by our global BC production estimate (based on Jones et al.^16^; see below), and uncertainties were computed in quadrature.

### BC production by landscape fires

Our estimate for the global BC production by landscape fires is based on the pyrogenic carbon production estimates of Jones et al.^16^ ($256_{ - 60}^{ + 84}$ Tg C year^−1^). First, we converted the nonparametric uncertainty in the global pyrogenic carbon production by landscape fires to a synthetic normal distribution bounded by the largest difference between the median and the upper and lower quartiles (±84 Tg C year^−1^). We present the uncertainty in the total pyrogenic carbon production flux as a 1*σ* uncertainty range, but note that the lower bound is conservative. Second, we calculated the global BC production flux as the product of the pyrogenic carbon production estimates and the BC content of pyrogenic carbon produced at a temperature range of 400–600 °C (50 ± 30%) as presented by Bird et al.^27^. For the uncertainty in global BC production, the 1*σ* uncertainties in the pyrogenic carbon production flux and the BC content of pyrogenic carbon were multiplied in quadrature.

## Supplementary information

Supplementary Information

Peer Review File

## Data Availability

The data set analysed and discussed in this article is provided as a Supplementary Dataset (SUPPLEMENTARY_DATASET.xlsx).

## Source data

Source Data
