# Supplementary material for: Fires prime terrestrial organic carbon for riverine export to the global oceans
Source: Nat Commun. 2020 Jun 3;11:2791. doi: 10.1038/s41467-020-16576-z (PMC7270114; doi:10.1038/s41467-020-16576-z)
Supplement: Supplementary file 1 — Supplementary Information [file 41467_2020_16576_MOESM1_ESM.pdf]

Supplementary Information for "Fires prime terrestrial organic carbon for riverine export to the global oceans" by Jones et al.

Supplementary Note 1: Is there a Globally Consistent Relationship between DBC and DOC Concentrations?

Following the addition of the new data to the global dataset (n = 409), we repeated the analysis of Jaffé et al. (ref. [1]) by re-fitting a simple linear model to concentrations of DBC and DOC. In accordance with the previous study, the relationship was fitted to the mean concentrations of DBC and DOC in 15 groups of samples that were binned according to their DOC concentration. This analysis suggested that the data conformed to a relationship given by the formula DBC = 0.102(DOC) – 0.12, which is close to the relationship reported originally by Jaffé et al. (ref. [1]; DBC = 0.106(DOC) – 0.09).

Although the predictive performance of the fitted model was high, we note that the binning approach is likely to have caused overestimation of explained variance by masking a portion of the variance present in the raw dataset. Moreover, the number of samples in each group was markedly higher in the "low DOC concentration" groups 1-5 (< 5 mg l^{-1}, n = 329) than in "high DOC concentration" groups 11-15 (> 9 mg l^{-1}, n = 17); therefore, the binning procedure disproportionately increased the leverage of the data points in the high DOC concentration groups, which predominantly represented samples from major polar rivers (n = 6) and minor rivers draining boreal forest (n = 4) and peatland (n = 3).

A coupled problem was that the variance in the DBC content of riverine DOC increased with DOC concentration, meaning that the binning procedure also disproportionately masks variance from the portions of the dataset with the greatest leverage over the fitted relationship. Nonetheless, from the perspective of robust regression model fitting, the binning procedure is necessary because the relationship fitted to raw data points would otherwise conform poorly to the assumption of residual heteroscedasticity. Taking into account these diagnostic challenges to statistical model fitting, we suggest the robustness of the fitted relationship would be aided by a greater number of observations from (sub)tropical and temperate rivers with high concentrations of DOC (e.g. > 9 mg l^{-1}).

Supplementary Note 2: Comparison of Different Flux Estimation Approaches

We evaluated whether global riverine DBC flux estimates are sensitive to the method of estimation where DBC is treated either as a globally uniform function of DOC concentration or as a spatially variable fraction of DOC concentration (Supplementary Table 2). Supplementary Figure 2 shows how the relationship between DBC and DOC concentrations is represented by these two contrasting *a priori* perspectives. In total, we consider 5 methods of global DBC flux estimation (Supplementary Table 2):

- method A is as presented by Jaffé et al. (ref. [1]), whereby the DBC content of DOC is predicted by a global relationship, fitted to their original dataset (n = 109), and the global DOC export flux estimated by Hedges et al. (ref. [2]; 250 Tg C year^{-1}; Supplementary Table 3);
- in method B, as an adjustment to method A, we incorporate more recent estimates for DOC fluxes from (sub)tropical (< 30° N/S; 128 ± 23 Tg C year^{-1}), temperate (30-60° N/S; 38 ± 4 Tg C year^{-1}) and high latitude (> 60° N/S; 39 ± 3 Tg C year^{-1}) rivers according to the meta-analysis of Dai et al. (ref. [3]; Supplementary Table 4);
- in method C, as a further adjustment to method B, we apply the predictive model that was re-fitted to the extended dataset presented herein (n = 409; Supplementary Table 5);
- in method D, we apply the average (± standard deviation) DBC content of DOC observed in all major global rivers (8.1 ± 4.5%; Supplementary Table 1) to the global estimate for DOC export of Dai et al. (ref. [3]; Supplementary Table 6), and;
- in method E, we apply the average (± standard deviation) values for the DBC content of DOC observed in major sub(tropical), temperate and high latitude rivers to the estimates of DOC export from these latitudinal bands provided by Dai et al. (ref. [3]; Supplementary Table 7).

Method E is the only method that diverges from the use of either **(i)** a single global relationship between DBC and DOC (as in methods A-C) or **(ii)** a single global value for the DBC content of DOC (as in method D). Method E has the advantage of using spatially explicit values for the contribution of DBC to riverine DOC and thus accounts for the systematic global variability in the DBC content of riverine DOC. The estimate for global DBC export that we present in this study derives from method E.

The global DBC export fluxes calculated using method A were significantly greater than those calculated using all other methods due to the significantly lower estimate for global

DOC export of Dai et al. (ref. [3]) than Hedges et al. (ref. [2]). Dai et al. (ref. [3]) meta-analysed global DOC concentration and river discharge data that was compiled over an additional ~15 years following the publication by Hedges et al. (ref. [2]) and, as a result of the richness of this dataset, were able to apportion global DOC fluxes to individual latitude bands. Differences in the global and latitudinal DBC export fluxes calculated according to methods B and C were minimal owing to the similarity of the models fitted to the original dataset of 109 data points compiled by Jaffé et al (ref. [1]) and to the extended global dataset of 409 data points compiled here (Supplementary dataset; summarised in Supplementary Table 1). There were minimal differences in global or latitudinal export fluxes between methods D and C, which is due to these methods relying on the same extended dataset of 409 DOC and DBC concentrations and their global approach to flux estimation; the regression line fitted to this dataset intersects close to the origin and thus the slope of the regression line is close to the mean DBC content of DOC.

The spatially variable method E, which we use as our estimate of global DBC export fluxes (see main text), produced an estimate for the global DBC flux of 18.0 ± 3.9 Tg C year^{-1} composed of 12.4 ± 3.8 Tg C year^{-1} from (sub)tropical rivers, 1.8 ± 0.6 Tg C year^{-1} from temperate rivers, and 3.8 ± 0.6 Tg C year^{-1} from high latitude rivers. The global DBC flux estimate deriving from method E was near to the value achieved by applying methods C and D, which were the most directly comparable to the method E as they utilised the same dataset of DOC and DBC concentrations and the same DOC export flux estimates (Supplementary Table 2).

Nonetheless, substantially different estimates for the DBC flux from individual latitudinal ranges were produced by the spatially variable approach (method E) and the spatially continuous approaches (methods C and D; Supplementary Table 2). The greatest absolute difference was observed in (sub)tropical latitudes, where the central estimate produced by approach E was 2.8 Tg C year^{-1} (22%) greater than the estimate produced by approach C and 2.0 Tg C year (16%) greater than the estimate produced by approach D. The central estimate produced by approach E for high-latitude export of DBC was also 0.6 Tg C year^{-1} (18%) greater than approach D and 0.4 Tg C year^{-1} (11%) greater than approach C. On the other hand, the central estimate for DBC export by temperate rivers produced by method E was 1.0 Tg C year^{-1} (35%) lower than in approach C and 1.3 Tg C year^{-1} (43%) lower than in approach D (Supplementary Table 2). These results indicate that DBC export fluxes are overestimated in temperate regions and underestimated in the (sub)tropics if a globally homogenous relationship between DBC and DOC concentrations is used to estimate

these fluxes. The differences in DBC flux estimates between the two methods were smaller in the high latitudes due to the disproportionate influence that high-latitude samples on the regression line fitted to DBC and DOC concentrations (Supplementary Note 1). This preordained that the mean contribution of DBC to DOC load for this region is closer to the global mean DBC content of DOC (Supplementary Figure 3).

Supplementary Tables

Supplementary Table 1: Mean (x̄) ± standard deviation (s) values for DOC concentrations, DBC concentrations and DBC/DOC ratios in the global dataset. The Δ values indicate the change in the number of studies (n) and x̄ compared to those in Jaffé et al. (ref. [1]) following inclusion of data from subsequently published studies (see methods)[4–11]. Classifications of major rivers derive from the Freshwater Ecoregions of the World (FEOW) dataset (ref. [12]), while minor channels draining specific land covers (not classified in the FEOW dataset) are classified as described in the primary literature[1,4–10,13].

		**Channel Count**		**Sample Count**		**DOC (mg carbon l^{-1})**			**DBC (mg carbon l^{-1})**			**DBC/DOC (%)**		
		**n**	**Δ**	**n**	**Δ**	**x̄**	**s**	**Δ**	**x̄**	**s**	**Δ**	**x̄**	**s**	**Δ**
						**Major Rivers by FEOW Ecoregion**								
High Latitude	Polar Freshwaters	6		26		8.43	3.90		0.83	0.41		9.6	1.5	
Temperate	Temperate Lowland	5	+1	26	+19	5.85	2.54	+2.3	0.31	0.10	+0.02	5.6	1.8	-2.1
	Temperate Upland & Lowland	5	+3	36	+34	1.97	0.62	-0.5	0.09	0.07	-0.08	4.2	2.2	-1.7
	All	10	+4	62	+53	3.60	2.56	+0.3	0.18	0.14	-0.08	4.8	2.2	-2.5
Tropical and Subtropical	(Sub)Tropical Lowland	9	+3	80	+73	2.18	2.03	-1.9	0.20	0.24	-0.23	9.2	2.3	+2.5
	(Sub)Tropical Upland	5	+4	19	+19	3.07	0.73	New	0.31	0.20	New	9.9	3.6	New
	(Sub)Tropical Upland & Lowland	3	+1	7	+5	4.98	1.62	+0.4	0.72	0.52	+0.28	14.5	8.2	+5.0
	All	17	+8	106	+97	2.52	1.97	-1.7	0.26	0.29	-0.18	9.7	3.4	+2.4
Xeric	Xeric	1		1		4.07			0.22			5.5		
						**Minor Channels**								
Minor Channels	Boreal Forest	11		11		6.33	6.61		0.39	0.46		5.4	1.1	
	Glaciers	6		6		0.67	0.23		0.02	0.01		2.1	1.0	
	Peatland	4		4		14.77	7.40		2.06	0.78		14.8	2.3	
	Savannah	38	+37	38	+38	1.45	0.67	New	0.20	0.06	New	15.2	5.0	New
	Temperate Forest	27	+15	55	+43	3.14	2.21	+1.6	0.14	0.12	+0.09	4.2	2.2	+0.2
	Temperate Grassland	11		11		1.69	0.31		0.06	0.02		3.3	0.6	
	Tropical Forest	39	+38	69	+69	1.95	1.18	New	0.15	0.09	New	8.6	3.3	New
	Wetland	15		20		6.02	3.07		0.51	0.25		8.5	2.5	
**All Global Measurements**		185	+102	409	+300	3.29	3.21	-2.0	0.27	0.34	-0.21	8.1	4.5	+1.0

Supplementary Table 2: Global riverine annual DBC export (Tg C year^{-1}) from (sub)tropical, temperate, high-latitude and all global rivers estimated according to the various calculation approaches discussed in the Supplementary Note 2.

Approach		(Sub)Tropical	Temperate	High Latitude	Global
**(i) A Single Global Relationship**					
**(A) Jaffé et al. (2013)**	DBC = 0.106(DOC); original global dataset of DOC and DBC concentrations; global DOC export = 250 Tg C year (Hedges et al., 1997). See Supplementary Table 4				26.5 (24.8 - 28.3)
**(B) A + updated global DOC export estimates**	DBC = 0.106(DOC) - 0.09; original global dataset of 109 DOC and DBC concentrations; global DOC export from Dai et al. (2012) See Supplementary Table 5	11.2 (7.4 - 15.1)	3.2 (2 - 4.4)	3.7 (3.1 - 4.4)	18.2 (15.2 - 21.2)
**(C) B + extended dataset**	DBC = 0.102(DOC) - 0.12; extended global dataset of 409 DOC and DBC concentrations; global DOC export from Dai et al. (2012) See Supplementary Table 6	9.7 (5.9 - 13.4)	2.7 (1.5 - 3.9)	3.4 (2.7 - 4.1)	15.8 (12.8 - 18.8)
**(ii) A Single Global DBC/DOC Ratio**					
**(D) This Study**	Global average DBC/DOC ratio for all MMRs (Table 1); extended global dataset of 409 DOC and DBC concentrations; global DOC export from Dai et al. (2012) See Supplementary Table 7	10.4 (4.3 - 16.5)	3.1 (1.3 - 4.9)	3.2 (1.4 - 5)	16.7 (11.5 - 21.9)
**(iii) Specific DBC/DOC Ratios for Each Latitude**					
**(E) This Study**	DBC/DOC ratios specific to MMRs in (sub)tropical, temperate and high latitude regions (Table 1); extended global dataset of DOC and DBC concentrations; global DOC export from Dai et al. (2012) See Supplementary Table 3	12.4 (7.5 - 17.3)	1.8 (0.9 - 2.6)	3.8 (3.1 - 4.4)	18.0 (14 - 21.9)
**Comparison of Spatially Variable and Spatially Continuous Approaches**					
**(E) – (C)**	Difference in central estimates	+2.8	-1	+0.4	+2.2
**(E) – (D)**	Difference in central estimates	+2	-1.3	+0.6	+1.3

Supplementary Table 3: Calculation of DBC fluxes according to method E, described in Supplementary Note 2. The mean (± 1 standard deviation) DOC concentrations derive from the meta-analysis of Dai et al. (ref. [3]). Estimated DOC export fluxes also derive from Dai et al. (ref. [3]) and the reported uncertainty ranges include uncertainty in water discharge rates and DOC concentrations. The DBC content of riverine DOC is specific to (sub)tropical, temperate and high latitude bands and is equal to the mean (± 1 standard deviation) value for the relevant major rivers included in the dataset. DBC fluxes are calculated as DBC/DOC × fDOC, where fDOC is the export flux for DOC. Uncertainty in DBC export fluxes is computed in quadrature and includes uncertainty in DOC export and the DBC content of riverine DOC.

Latitudinal Range		DOC Export Flux (Tg C year^{-1})				DBC/DOC (%)		DBC Export Flux (Tg C year^{-1})			
		Central		± 1 St. Dev.		Central	± 1 St. Dev.	Central		± 1 St. Dev.	
**(Sub)tropical**	0-30N	38.0	128.0	2.6	20.4	9.7%	3.4%	3.7	12.4	1.3	3.9
	0-30S	90.0		20.2				8.7		3.6	
**Temperate**	30-60N	30.3	38.0	0.9	3.5	4.8%	2.2%	1.5	1.8	0.7	0.7
	30-60S	7.7		3.4				0.4		0.2	
**High Latitude**	>60N	39.3	39.3	3.0	3.0	9.6%	1.5%	3.8	3.8	0.6	0.6
**Global**		205.3		20.9				18.0		4.0	

Supplementary Table 4: Calculation of DBC fluxes according to method A, following Jaffé et al. (ref. [1]) as described in Supplementary Note 2. The estimate for the global DOC export derives from Hedges et al. (ref. [2]). The DBC content of riverine DOC is calculated as (DOC × m), where DOC is the concentration of DOC and m is the slope of the regression line ± the standard error of the fitted slope (0.106 ± 0.007). The intercept term of the regression equation is ignored. Estimates of the DBC content of DOC are based on 109 data points, grouped into 15 classes according to their DOC concentration. DBC fluxes are calculated as DBC/DOC × fDOC, where fDOC is the export flux for DOC. Uncertainty in the DBC content of riverine DOC is propagated through this calculation step; uncertainty in the global DOC export flux is not included.

Latitudinal Range	DBC/DOC (%)		DOC Export Flux (Tg C year^{-1})		DBC Flux (Tg C year^{-1})	
	Mean	± Uncertainty	Central	± Uncertainty	Central	± Uncertainty
**Global**	0.106	0.007	250	N/A	26.5	1.7

Supplementary Table 5: Calculation of DBC fluxes according to method B, as described in Supplementary Note 2. The mean (± 1 standard deviation) DOC concentrations derive from the meta-analysis of Dai et al. (ref. [3]). Estimated DOC export fluxes also derive from Dai et al. (ref. [3]) and the reported uncertainty ranges include uncertainty in water discharge rates and DOC concentrations. The DBC content of riverine DOC is calculated as ((DOC × *m*) + *c*)/DOC, where DOC is the concentration of DOC, *m* is the slope of the regression equation (0.106 ± 0.007) and *c* is the intercept (-0.09 ± 0.07). Estimates of the DBC content of DOC are based on 109 data points, grouped into 15 classes according to their DOC concentration. Uncertainty in the DBC content of riverine DOC includes standard error in regression model coefficients and standard deviation in observed DOC concentrations. DBC fluxes are calculated as DBC/DOC × fDOC, where fDOC is the export flux for DOC. Uncertainty in DBC export fluxes is computed in quadrature and includes uncertainty in DOC export and the DBC content of riverine DOC.

**Latitudinal Range**		**DOC Concentration (mg l^{-1})**		**DBC/DOC (%)**		**DOC Export Flux (Tg C $year^{-1}$)**				**DBC Flux (Tg C $year^{-1}$)**			
		**Mean**	**± 1 St. Dev.**	**Predicted**	**± Uncertainty**	**Central**		**± Uncertainty**		**Central**		**± Uncertainty**	
**(Sub)tropical**	0-30N	6.35	1.43	9.2%	3.4%	38.0	128.0	2.6	20.4	3.5	11.2	1.3	2.8
	0-30S	4.27	0.29	8.6%	2.0%	90.0		20.2		7.7		2.5	
**Temperate**	30-60N	3.87	0.12	8.4%	2.0%	30.3	38.0	0.9	3.5	2.5	3.2	0.6	0.8
	30-60S	5.20	2.32	8.9%	6.4%	7.7		3.4		0.7		0.6	
**High Latitude**	>60N	8.02	0.60	9.5%	1.5%	39.3	39.3	3.0	3.0	3.7	3.7	0.7	0.7
**Global**						205.3		20.9		18.2		3.0	

Supplementary Table 6: Calculation of DBC fluxes according to method C, as described in Supplementary Note 2. The mean (± 1 standard deviation) DOC concentrations derive from the meta-analysis of Dai et al. (ref. [3]). Estimated DOC export fluxes also derive from Dai et al. (ref. [3]) and the reported uncertainty ranges include uncertainty in water discharge rates and DOC concentrations. The DBC content of riverine DOC is calculated as ((DOC × *m*) + *c*)/DOC, where DOC is the concentration of DOC, *m* is the slope of the regression equation (0.102 ± 0.008) and c is the intercept (-0.123 ± 0.08). Estimates of the DBC content of DOC are based on 409 data points, grouped into 15 classes according to their DOC concentration. Uncertainty in the DBC content of riverine DOC includes standard error in regression model coefficients and standard deviation in observed DOC concentrations. DBC fluxes are calculated as DBC/DOC × fDOC, where fDOC is the export flux for DOC. Uncertainty in DBC export fluxes is computed in quadrature and includes uncertainty in DOC export and the DBC content of riverine DOC.

**Latitudinal Range**		**DOC Concentration (mg l^{-1})**		**DBC/DOC (%)**		**DOC Export Flux (Tg C $year^{-1}$)**				**DBC Flux (Tg C $year^{-1}$)**			
		**Mean**	**± 1 St. Dev.**	**Predicted**	**± Uncertainty**	**Central**		**± Uncertainty**		**Central**		**± Uncertainty**	
**(Sub)tropical**	0-30N	6.35	1.43	8.2%	3.3%	38.0	128.0	2.6	20.4	3.1	9.7	1.3	2.8
	0-30S	4.27	0.29	7.3%	2.2%	90.0		20.2		6.5		2.5	
**Temperate**	30-60N	3.87	0.12	7.0%	2.3%	30.3	38.0	0.9	3.5	2.1	2.7	0.7	0.9
	30-60S	5.2	2.32	7.8%	6.0%	7.7		3.4		0.6		0.5	
**High Latitude**	>60N	8.02	0.6	8.6%	1.6%	39.3	39.3	3.0	3.0	3.4	3.4	0.7	0.7
**Global**						205.3		20.9		15.8		3.0	

Supplementary Table 7: Calculation of DBC fluxes according to method D, as described in Supplementary Note 2. The mean (± 1 standard deviation) DOC concentrations derive from the meta-analysis of Dai et al. (ref. [3]). Estimated DOC export fluxes also derive from Dai et al. (ref. [3]) and the reported uncertainty ranges include uncertainty in water discharge rates and DOC concentrations. The DBC content of riverine DOC is constant for all latitudinal bands and is equal to the mean (± 1 standard deviation) value for all major rivers included in the dataset. DBC fluxes are calculated as DBC/DOC × fDOC, where fDOC is the export flux for DOC. Uncertainty in DBC export fluxes is computed in quadrature and includes uncertainty in DOC export and the DBC content of riverine DOC.

<table>
<tr><th colspan="2" rowspan="2">Latitudinal Range</th><th colspan="4">DOC Export Flux (Tg C year^{-1})</th><th colspan="2">DBC/DOC (%)</th><th colspan="4">DBC Export Flux (Tg C year^{-1})</th></tr>
<tr><th colspan="2">Central</th><th colspan="2">± 1 St. Dev.</th><th>Central</th><th>± 1 St. Dev.</th><th colspan="2">Central</th><th colspan="2">± 1 St. Dev.</th></tr>
<tr><td rowspan="2">(Sub)tropical</td><td>0-30N</td><td>38.0</td><td rowspan="2">128.0</td><td>2.6</td><td rowspan="2">20.4</td><td rowspan="5">8.1%</td><td rowspan="5">4.5%</td><td>3.1</td><td rowspan="2">10.4</td><td>1.7</td><td rowspan="2">4.7</td></tr>
<tr><td>0-30S</td><td>90.0</td><td>20.2</td><td>7.3</td><td>4.3</td></tr>
<tr><td rowspan="2">Temperate</td><td>30-60N</td><td>30.3</td><td rowspan="2">38.0</td><td>0.9</td><td rowspan="2">3.5</td><td>2.5</td><td rowspan="2">3.1</td><td>1.4</td><td rowspan="2">1.4</td></tr>
<tr><td>30-60S</td><td>7.7</td><td>3.4</td><td>0.6</td><td>0.4</td></tr>
<tr><td>High Latitude</td><td>>60N</td><td>39.3</td><td>39.3</td><td>3.0</td><td>3.0</td><td>3.2</td><td>3.2</td><td>1.8</td><td>1.8</td></tr>
<tr><td>Global</td><td></td><td colspan="2">205.3</td><td colspan="2">20.9</td><td colspan="2"></td><td colspan="2">16.7</td><td colspan="2">5.2</td></tr>
</table>

Supplementary Figures

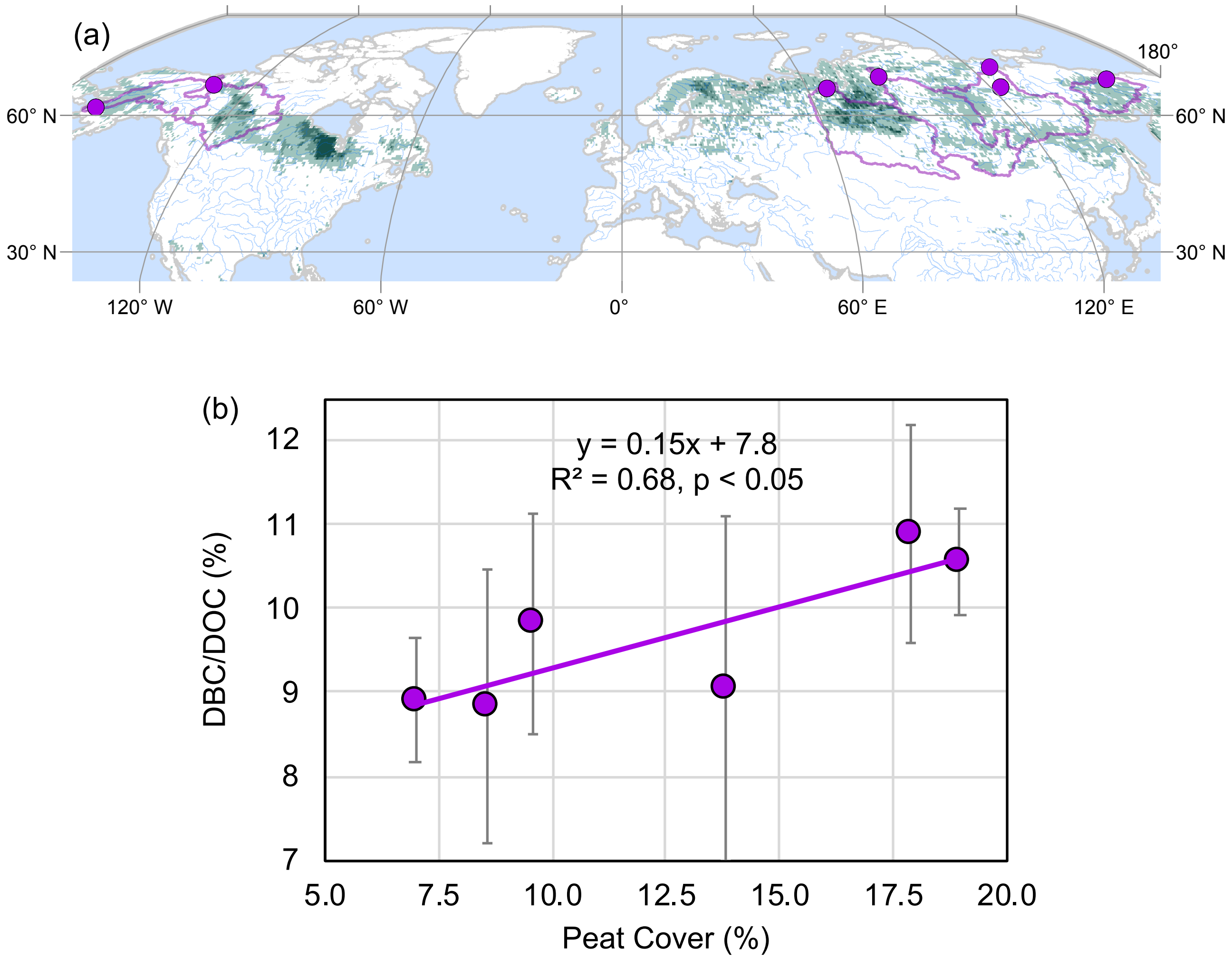

Supplementary Figure 1: (a) Peatland cover in the mid-to-high northern latitudes. Data derives from Earth system model predictions of peatland cover validated using a qualitative presence/absence product (ref. [14]). Points mark sampling locations as in Figure 2 and represent the 6 major high-latitude rivers (Kolyma, Lena, Mackenzie, Ob, Yenisey, Yukon). **(b)** Scatter plot showing the average DBC content of DOC in the 6 major high-latitude rivers across replicated seasonal samples, as well as the average peatland cover within the catchment of each major river. The error bars plot the standard deviation in DBC content of DOC across replicated seasonal samples. For the Lena River, one additional sample was collected upstream of the estuary and this was also included in the average. The simple linear relationship between the DBC content of riverine DOC and peatland cover is also plotted.

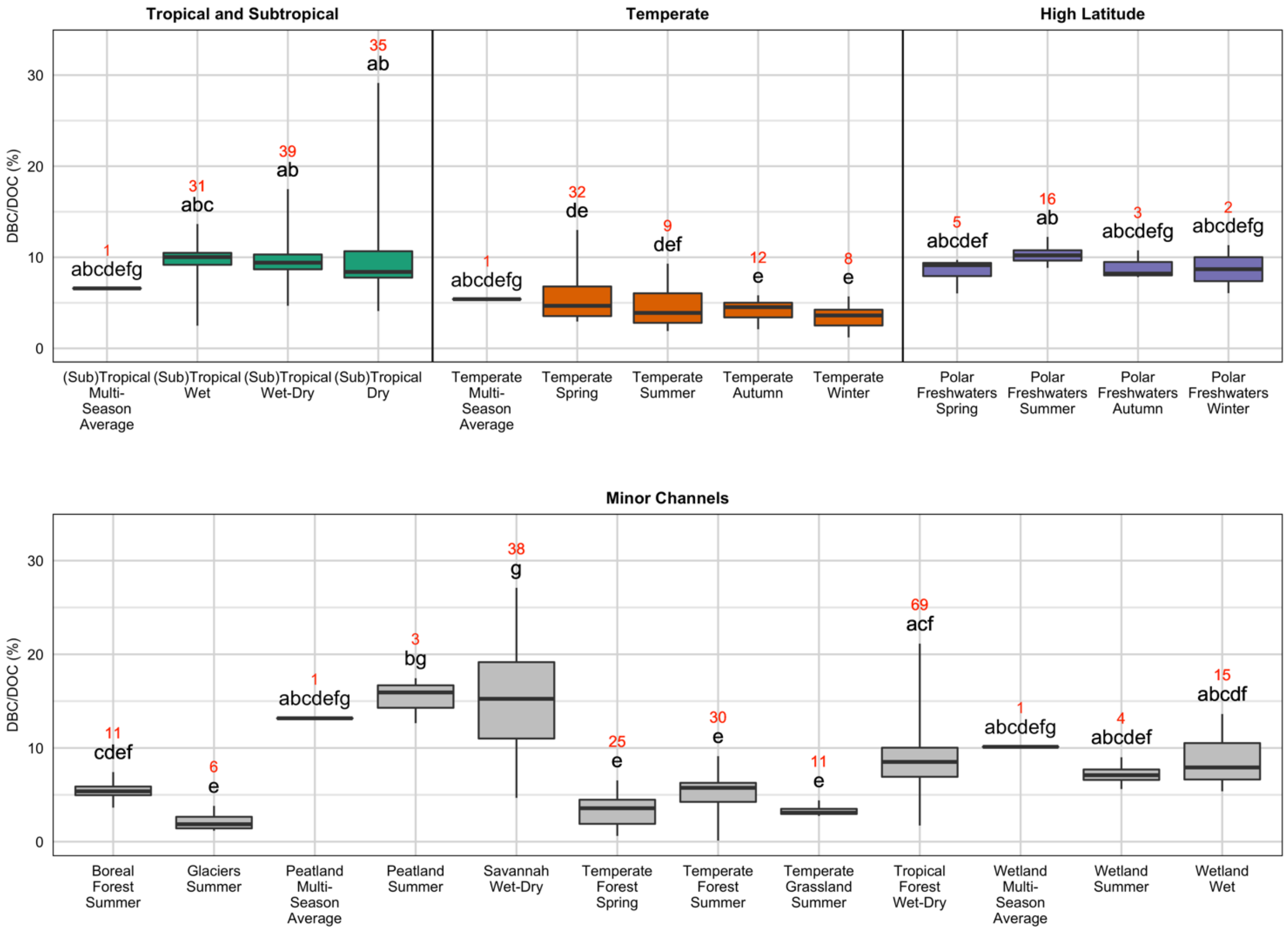

Supplementary Figure 2: A seasonal breakdown of the distribution of the DBC content of DOC (%) in major rivers and minor channels. Bold horizontal lines within the boxes represent median values, boxes represent interquartile ranges, and whiskers mark full ranges. The timing of the collection of (sub)tropical samples is classified as follows: wet season (Wet), wet-to-dry season transition (Wet-Dry), dry season (dry) or dry-to-wet season transition (Dry-Wet). The timing of the collection of extra-tropical samples is classified as follows for Northern Hemisphere samples: Spring (March-May), Summer (June-August), Autumn (September-November) or Winter (December-February). The equivalent (inverse) Austral seasons are used for Southern Hemisphere. The four data points marked as "Multi-Season Average" represent the average of multiple samples collected in the same channel across multiple seasons. Red numbers indicate the number of data points included in each channel class. Letters denote groups with statistically similar mean values according to a Tukey honest significant difference (HSD) test (see methods).

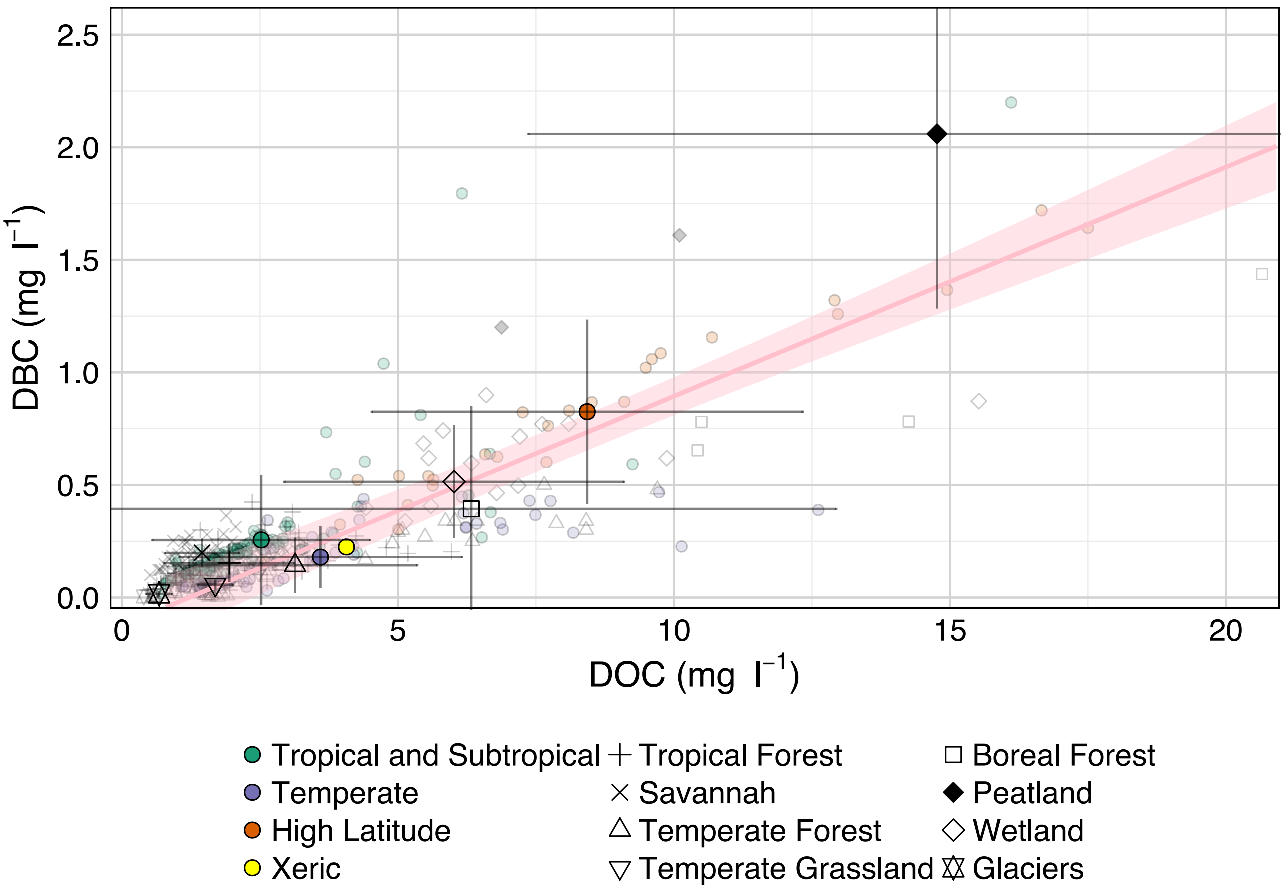

Supplementary Figure 3: A scatter plot of DBC and DOC concentrations represented in the global dataset. Translucent points in the background plot each data point in the global dataset. Larger, opaque points in the foreground plot the average values for points in each category of channel. Error bars emanating from these average points represent the standard deviation in concentrations in each category of channel. The red line and shaded range show the global relationship fitted to all data points (DBC = 0.102(DOC) - 0.12) and the standard error of the prediction, respectively.

Supplementary References

1. Jaffé, R. *et al.* Global charcoal mobilization from soils via dissolution and riverine transport to the oceans. *Science* **340**, 345–7 (2013).

2. Hedges, J. I., Keil, R. G. & Benner, R. What happens to terrestrial organic matter in the ocean? *Org. Geochem.* **27**, 195–212 (1997).

3. Dai, M., Yin, Z., Meng, F., Liu, Q. & Cai, W.-J. Spatial distribution of riverine DOC inputs to the ocean: an updated global synthesis. *Curr. Opin. Environ. Sustain.* **4**, 170–178 (2012).

4. Jones, M. W. *et al.* Environmental Controls on the Riverine Export of Dissolved Black Carbon. *Global Biogeochem. Cycles* 2018GB006140 (2019). doi:10.1029/2018GB006140

5. Roebuck, J. A., Seidel, M., Dittmar, T. & Jaffe, R. Land Use Controls on the Spatial Variability of Dissolved Black Carbon in a Subtropical Watershed. *Environ. Sci. Technol.* acs.est.8b00190 (2018). doi:10.1021/acs.est.8b00190

6. Wagner, S., Cawley, K. M., Rosario-Ortiz, F. L. & Jaffé, R. In-stream sources and links between particulate and dissolved black carbon following a wildfire. *Biogeochemistry* **124**, 145–161 (2015).

7. Wang, X., Xu, C., Druffel, E. M., Xue, Y. & Qi, Y. Two black carbon pools transported by the Changjiang and Huanghe Rivers in China. *Global Biogeochem. Cycles* **30**, 1778–1790 (2016).

8. Marques, J. S. J. *et al.* Dissolved Black Carbon in the Headwaters-to-Ocean Continuum of Paraíba Do Sul River, Brazil. *Front. Earth Sci.* **5**, 1–12 (2017).

9. Coppola, A. I. *et al.* Marked isotopic variability within and between the Amazon River and marine dissolved black carbon pools. *Nat. Commun.* 1–8 (2019). doi:10.1038/s41467-019-11543-9

10. Bao, H., Niggemann, J., Huang, D., Dittmar, T. & Kao, S. Different Responses of Dissolved Black Carbon and Dissolved Lignin to Seasonal Hydrological Changes and an Extreme Rain Event. *J. Geophys. Res. Biogeosciences* **124**, 479–493 (2019).

11. Mannino, A. & Harvey, H. R. Black carbon in estuarine and coastal ocean dissolved organic matter. *Limnol. Oceanogr.* **49**, 735–740 (2004).

12. Abell, R. *et al.* Freshwater Ecoregions of the World: A New Map of Biogeographic Units for Freshwater Biodiversity Conservation. *Bioscience* (2008).

doi:10.1641/B580507

13. Roebuck, J. A., Medeiros, P. M., Letourneau, M. L. & Jaffé, R. Hydrological Controls on the Seasonal Variability of Dissolved and Particulate Black Carbon in the Altamaha River, GA. *J. Geophys. Res. Biogeosciences* **123**, 3055–3071 (2018).

14. Wu, Y., Chan, E., Melton, J. R. & Verseghy, D. L. A map of global peatland distribution created using machine learning for use in terrestrial ecosystem and earth system models. *Geosci. Model Dev.* 1–21 (2017). doi:10.5194/gmd-2017-152
