# Supplementary material for: Fires prime terrestrial organic carbon for riverine export to the global oceans
Source: Nat Commun. 2020 Jun 3;11:2791. doi: 10.1038/s41467-020-16576-z (PMC7270114; doi:10.1038/s41467-020-16576-z)
Supplement: Supplementary file 2 — Peer Review File [file 41467_2020_16576_MOESM2_ESM.docx]

Peer Review Information

**Journal:** Nature Communications

**Manuscript title:** Fires prime terrestrial organic carbon for riverine export to the global oceans

**Corresponding author name(s):** Dr Matthew James

**Editorial notes:**

**Transferred manuscripts**

This manuscript has been previously reviewed at another journal that is not operating a transparent peer review scheme. This document only contains reviewer comments and rebuttal letters for versions considered at *Nature Communications*.

**Transferred manuscripts (no peer review at *Nature Communications*)**

This manuscript has been previously reviewed at another journal that is not operating a transparent peer review scheme. The manuscript was considered suitable for publication without further review at *Nature Communications*.

**Redactions – transferred manuscripts (mention of previous referee reports from elsewhere)**

This manuscript has been previously reviewed at another journal that is not operating a transparent peer review scheme. This document only contains reviewer comments and rebuttal letters for versions considered at *Nature Communications*. Mentions of prior referee reports have been redacted.

**Redactions – transferred manuscripts (mention of the other journal)**

This manuscript has been previously reviewed at another journal that is not operating a transparent peer review scheme. This document only contains reviewer comments and rebuttal letters for versions considered at *Nature Communications*. Mentions of the other journal have been redacted.

**Redactions – unpublished data**

Parts of this Peer Review File have been redacted as indicated to maintain the confidentiality of unpublished data.

**Redactions – confidential patient information**

Parts of this Peer Review File have been redacted as indicated to maintain patient confidentiality.

**Redactions – published data**

Parts of this Peer Review File have been redacted as indicated to remove third-party material where no permission to publish could be obtained.

**Amendments – published data**

[Figure X/The figure on page X] in this Peer Review File has been amended to remove third-party material where no permission to publish could be obtained.

**Published data (credit line for image)**

[Figure X/The figure on page X] in this Peer Review File is reproduced with permission from [Copyright Holder].

**Redactions – reviewer opt-out**

Parts of this Peer Review File have been redacted as indicated as we could not obtain permission to publish the reports of reviewer ###.

**Reviewer comments in marked-up manuscript**

In their review of the [first/second/third/...] version of this manuscript, reviewer ### added their comments to the manuscript file. These comments, excluding minor textual revisions, have been copied into this Peer Review File.

**Reviewer comments & decisions:**

**Reviewer comments, first version:**

Reviewers' comments:

Reviewer #1 (Remarks to the Author):

The production of black carbon (BC) and its transport to, and deep burial within, the global oceans is an uncertain component of carbon residence time in Earth System models because the degradation of BC is inherently slower than that of other organic matter and its fate in the ocean is poorly understood. This work builds on that of Jaffe et al. (2013) in examining global BC mobilization from soil to stream to ocean, and adds a significant number of new sites from broad geographical provinces. The analyses appear to be sound. I found the findings to be important and offer very minor comments, below, and hope they are helpful in minor revision.

Regarding the introduction, and later in the analyses, the issue of re-burning of BC should be addressed. Some pyrogenic carbon in the terrestrial and soil pools is consumed in subsequent wildfires, and thus does not contribute to the pool of BC available to leach or mobilize into the DBC pool. There are papers that discus the re-burning mechanism and specifically why BC does not always accumulate in the mineral soil in fire prone ecosystems, and I think it would be valuable to briefly mention this.

Regarding “Black carbon content of riverine dissolved organic carbon”-

A large part of this analysis and discussion is on percent export, of the carbon that is being exported. Figure 1 describes fluxes of transport via arrows, but there are no magnitudes associated with them. It would be better if this could be integrated with Figure 2 in some better way. Maybe the arrows in Figure 1 could have weight associated with magnitude flux?

Regarding “Global Riverine Export of Black Carbon”-

The estimate that rivers export at total of 43+/-15 Tg BC/yr has pretty huge error/uncertainty. What are the biggest sources of error here? What research is needed to constrain this?


Reviewer #2 (Remarks to the Author):

A) Overview
This is a very interesting paper, by a team that continues to advance the understanding of the production and cycling of black carbon (BC) in the environment. The authors present a revision of global inputs of dissolved black carbon (DBC, characterized as the highly condensed portion of the pyrogenic organic carbon continuum) to the ocean. The major revision comes from a) a large increase of the data set used in the modeling (from ~110 to >410 data points) and b) a discrimination of inputs based on large global biomes. The overall conclusions are equally twofold: a) a demonstration of a large-scale geographical heterogeneity in DBC fluxes to the ocean, which results in b) a significant correction of the estimates of global oceanic DBC fluxes. Their most significant contribution to carbon cycling maybe lies in the suggestion (remaining to be tested and confirmed) that once altered by fire, terrestrial pyrogenic organic carbon may be exported towards the ocean at a greater rate than general TOC in watersheds, thus lengthening the residence time of TOC in the environment. Considering the expected changing fire incidence and intensity conditions in coming decades, this may be a critical process to integrate into global biogeochemical models of carbon.
In the present manuscript, the authors clarify, and estimate, the geographical variability of the DBC flux providing very valuable adjustments to the prior estimates of global DBC fluxes. However, their study does not evaluate, or even mention, the potential influence of seasonality on DBC fluxes. The assumption that DBC/DOC ratios remain constant over the year and that annual DBC fluxes for any river can be modeled by knowing the DOC fluxes and selected DBC/DOC values is over simplified. This is not a fatal flaw of the study as the authors have demonstrated that the understanding of BC production and cycling in the environment is still evolving. We need such studies to better define the incremental gains in this understanding while at the same time identifying the areas of uncertainty that need constraining. How can the authors estimate the temporal variability in global fluxes? For example, a discussion on how seasonality is integrated into the river DOC fluxes is critical to understand the potential errors in flux estimates (DOC, and consequently DBC, fluxes can vary by orders of magnitude between base flow and peak flow periods). Secondly, prior work (Myers-Pigg et al. 2017), suggests that the low-temperature fraction of the DCB may not respond linearly to DOC hydrographic response. Some discussion needs to address this potential issue. For example, what proportion of the total DBC may occur in the low-T part of the spectrum? The CTO method used in this study only recovers the highly condensed, refractory component of the pyrogenic carbon pool, and this fraction seems to respond rather linearly to the DOC concentrations and hydrographic variations. However, the BPCS method also used extracts pyrogenic constituents that are produced at mid-range temperatures (down to ~300C), which suggests that some functionality exists in these fractions leading to solubility and transport behaviors comparable to low-temperature pyrogenic carbon pools. This incorporates a level of uncertainty in the flux estimates that needs to be addressed, if only qualitatively. Some questions to address could include the following: How many of the samples are taken early, during, late in the peak of the streamflow and which are taken during baseflow periods. What is a potential added source of error due to temporal variability and decoupling of different DCB fractions with DOC suggests
The quality of the sample set, data, and interpretations in this manuscript justify its acceptation and publication in Nature Communications. It is very well written and I have really no comments on its form. My recommendation is “accept with minor revisions”. I suggest that the manuscript would benefit from clarifications of some sources of errors due to temporal variability of DOC and DBC fluxes in major rivers along with potential temporal variabilities in DBC/DOC ratios.

B) Specific Comments
Please find an annotated pdf file attached to this review. The summary of all annotations appears at the head of the paper.


Reviewer #3 (Remarks to the Author):

Review comments on manuscript #NCOMMS-20-00624-T
entitled: Fires prime terrestrial organic carbon for riverine export to the global oceans.

The manuscript, from Jones et al., reports estimates of latitudinal- and biome-dependent dissolved black carbon (DBC) exports from rivers into oceans. These estimates were based on meta-analysis of an expanded dataset of 409 observations compiled from published and unpublished data. The authors concluded that both variability across latitude and biome were significant - with rivers in the tropics and high latitude discharging higher amounts of dissolved black carbon to the ocean, than those in temperate latitudes. Though not qualitatively surprising, the authors’ findings provide some semi-quantitative estimates for spatially constraining DBC riverine-ocean flux. There are however, a series of ambiguities that prevents the reviewer from recommending this manuscript for publication without substantial revisions. The major ambiguities are provided below:

1. Beside the reference years, the timing/temporal criteria for a sample to be in the database is not clear. Were the meta-analysis criteria for rivers sampled at a comparable stage (e.g. flood stage)? Were the samples distributed across the year or confined to a given season (e.g. fire or otherwise)? In other words, how does the reviewer ascertain that the spatial (latitudinal and biome) differences reported were not really artefacts of temporally biased sampling arising from incomparable timing (e.g. in dry vs. wet or fire vs. non-fire seasons)?
2. It is not clear in the main manuscript what 409 observations meant. Are these from 5, 10, 15 rivers? Across 1, 3, 5 years? How do we ascertain that beyond just having a larger n than the incorporated databases, the new “expanded” database has more value? The authors need to clarify and clearly establish why their addition to, and aggregation or disaggregation of, this data to address their questions on DBC flux is valid. Although the authors pointed to the use of non-parametric statistics, such analyses still require standards. It was not clear to the reviewer that these standards were met.
3. Beyond putting some early global estimates on the long suspected and logical biome-dependent riverine DBC flux; the novelty of the methods and findings are lacking. Even with using a different, more complicated statistical approach to estimate global riverine-ocean DBC flux, the results (in essence) confirmed the previously simpler determined estimates of Jaffe et al.
4. The non-DBC sections of the manuscript were at best, hand-waving and really did not add anything to current understanding of BC cycling between terrestrial and oceanic systems. These sections which incidentally made up most of the paper lack a systematic, robust, analytical assessment and more often, than not, felt like a mad dash of grabbing numbers and combining them into a back-of-the-envelope calculation with minimal regard for scientific rigor. This led to a manuscript that started out reasonably well and focused but quickly descended into a frantic quest for plugging “global” gaps outside the scope of the dataset at the expense of developing a robust paper around a single theme.
5. Figure 1 is beautiful! The reviewer loves it. In the light of authors discussion on BC being more persistent than non-BC, how do they rationalize the rather small fraction of PBC in terrigenous POC?

**Author rebuttal, first version:**

**Reviewer #1 (Remarks to the Author):**

The production of black carbon (BC) and its transport to, and deep burial within, the global oceans is an uncertain component of carbon residence time in Earth System models because the degradation of BC is inherently slower than that of other organic matter and its fate in the ocean is poorly understood. This work builds on that of Jaffe et al. (2013) in examining global BC mobilization from soil to stream to ocean, and adds a significant number of new sites from broad geographical provinces. The analyses appear to be sound. I found the findings to be important and offer very minor comments, below, and hope they are helpful in minor revision.

Many thanks for your positive and constructive review.

Regarding the introduction, and later in the analyses, the issue of re-burning of BC should be addressed. Some pyrogenic carbon in the terrestrial and soil pools is consumed in subsequent wildfires, and thus does not contribute to the pool of BC available to leach or mobilize into the DBC pool. There are papers that discus the re-burning mechanism and specifically why BC does not always accumulate in the mineral soil in fire prone ecosystems, and I think it would be valuable to briefly mention this.

A restructured paragraph of the introduction now includes reference to the re-combustion of BC (please see below).

The BC produced by landscape fires has three potential fates. First, it can be mineralised through biotic or abiotic processes^20,21^, such as microbial decomposition ***or combustion during subsequent fires*^22,23^**. Second, it can reach deep soil stores where its decomposition is exceptionally limited^24,25^. Third, it can be exported to the global oceans as a fraction of riverine dissolved organic carbon (DOC) or particulate organic carbon (POC)^26,27^.

Regarding “Black carbon content of riverine dissolved organic carbon”-

A large part of this analysis and discussion is on percent export, of the carbon that is being exported. Figure 1 describes fluxes of transport via arrows, but there are no magnitudes associated with them. It would be better if this could be integrated with Figure 2 in some better way. Maybe the arrows in Figure 1 could have weight associated with magnitude flux?

In Figure 1, the dimensions of the triangles below “erosion of particulates” and “mobilisation of solutes” varies with the flux magnitude (see key to the left).

Regarding “Global Riverine Export of Black Carbon”-

The estimate that rivers export at total of 43+/-15 Tg BC/yr has pretty huge error/uncertainty. What are the biggest sources of error here? What research is needed to constrain this?

We have added discussion of the main sources of uncertainty to the main text, as follows:

“The uncertainty range of our DBC export estimate represents our best estimate of 1σ uncertainty (i.e. we consider there to be a 68% likelihood of the export fluxes falling within the stated bounds; see methods). The 1σ uncertainty of the DBC content of DOC is 35% in (sub)tropical samples, 43% in the temperate samples and 15% in high latitude samples (Figure 3). Meanwhile, the 1σ uncertainty of global DOC export fluxes is 16% for (sub)tropical

1

rivers, 9% for temperate rivers and 8% for high latitude samples (Supplementary Table S7). The moderately high uncertainty in the DBC content of DOC and the DOC export flux in the (sub)tropics, combined with the large contribution of tropical rivers to global DOC export, means that most of uncertainty in the global DBC export flux derives from the (sub)tropical flux. For our global estimate of total BC export, most of the global uncertainty derives from the existing estimate of PBC export, whose 1σ uncertainty exceeds 50%.”

2

**Reviewer #2 (Remarks to the Author):
*A) Overview***

This is a very interesting paper, by a team that continues to advance the understanding of the production and cycling of black carbon (BC) in the environment. The authors present a revision of global inputs of dissolved black carbon (DBC, characterized as the highly condensed portion of the pyrogenic organic carbon continuum) to the ocean. The major revision comes from a) a large increase of the data set used in the modeling (from ~110 to >410 data points) and b) a discrimination of inputs based on large global biomes. The overall conclusions are equally twofold: a) a demonstration of a large-scale geographical heterogeneity in DBC fluxes to the ocean, which results in b) a significant correction of the estimates of global oceanic DBC fluxes. Their most significant contribution to carbon cycling maybe lies in the suggestion (remaining to be tested and confirmed) that once altered by fire, terrestrial pyrogenic organic carbon may be exported towards the ocean at a greater rate than general TOC in watersheds, thus lengthening the residence time of TOC in the environment. Considering the expected changing fire incidence and intensity conditions in coming decades, this may be a critical process to integrate into global biogeochemical models of carbon.

Many thanks for your positive and constructive review.

In the present manuscript, the authors clarify, and estimate, the geographical variability of the DBC flux providing very valuable adjustments to the prior estimates of global DBC fluxes. However, their study does not evaluate, or even mention, the potential influence of seasonality on DBC fluxes. The assumption that DBC/DOC ratios remain constant over the year and that annual DBC fluxes for any river can be modeled by knowing the DOC fluxes and selected DBC/DOC values is over simplified. This is not a fatal flaw of the study as the authors have demonstrated that the understanding of BC production and cycling in the environment is still evolving. We need such studies to better define the incremental gains in this understanding while at the same time identifying the areas of uncertainty that need constraining. How can the authors estimate the temporal variability in global fluxes? For example, a discussion on how seasonality is integrated into the river DOC fluxes is critical to understand the potential errors in flux estimates (DOC, and consequently DBC, fluxes can vary by orders of magnitude between base flow and peak flow periods).

Secondly, prior work (Myers-Pigg et al. 2017), suggests that the low-temperature fraction of the DCB may not respond linearly to DOC hydrographic response. Some discussion needs to address this potential issue. For example, what proportion of the total DBC may occur in the low-T part of the spectrum? The CTO method used in this study only recovers the highly condensed, refractory component of the pyrogenic carbon pool, and this fraction seems to respond rather linearly to the DOC concentrations and hydrographic variations. However, the BPCS method also used extracts pyrogenic constituents that are produced at mid-range temperatures (down to ~300C), which suggests that some functionality exists in these fractions leading to solubility and transport behaviors comparable to low-temperature pyrogenic carbon pools. This incorporates a level of uncertainty in the flux estimates that needs to be addressed, if only qualitatively. Some questions to address could include the following: How many of the samples are taken early, during, late in the peak of the streamflow and which are taken during baseflow periods. What is a potential added source of error due to temporal variability and decoupling of different DCB fractions with DOC suggests

Thanks for this thought-provoking comment. We agree that seasonality is most likely to impact the more labile fractions of the pyrogenic carbon continuum, including some fractions that are detected with the BPCA approach. Consequently, we also agree that it was important to test whether bias in the timing of sampling influences the spatial patterns of the DBC content of DOC that we observed in our dataset. Please see our response to the comment immediately below for full details of our seasonal bias testing and results (and for an express overview, please see Figure S3).

3

In addition, we note the importance to be more explicit about which fractions of the pyrogenic carbon continuum are included in our estimates, and which fractions are not, and so we added the following paragraph to the main text:

“Our estimates account for the organic products of fire whose recalcitrance is considered to differ substantially from that of unburned vegetation (see methods). We do not account for the export of other soluble products of low-temperature vegetation charring, such as levoglucosan, which have short residence times compared to those of the DBC and thus lesser significance for the oceanic storage of terrigenous carbon^40,76,77^.”

Although we do not further discuss which pyrogenic carbon fractions are more/less susceptible to seasonal bias, we do verify that significant seasonality in the DBC content of DOC is not present in our dataset. We hope that the two lines of revision discussed above can jointly serve to alleviate the concern of the reviewer: that is, that BPCA-detected fractions of DBC have significant seasonal dependency.

The quality of the sample set, data, and interpretations in this manuscript justify its acceptation and publication in Nature Communications. It is very well written and I have really no comments on its form. My recommendation is “accept with minor revisions”. I suggest that the manuscript would benefit from clarifications of some sources of errors due to temporal variability of DOC and DBC fluxes in major rivers along with potential temporal variabilities in DBC/DOC ratios.

Thanks for your constructive feedback, particularly on the issue of seasonality/temporal variability of DBC/DOC ratios. We also note that most of the comments from the attached PDF: 1-1, 3-1, 5-1, 6-1, 9-2, 10-1, 11-1 also relate to this topic, so we provide here a response that seeks to address the collective points raised.

We agree that it is important to quantify the extent to which seasonal/temporal variability in DBC/DOC %, combined with the timing of sample collection, influences the spatial patterns we observe in our dataset.

As such we have taken the following steps:

1. We incorporated temporal sampling information (season) into our dataset of DBC and DOC concentration measurements.
2. For studies with multiple sampling periods, we used Tukey HSD tests to identify instances where there was variability across seasons within river classes.
3. We present the results of the above analyses in the supplementary material – please see Supplementary Figure S3.
4. We incorporate the following paragraph into the main text:

The dataset on which our global export estimates are based captures global-scale spatial variability in the DBC content of riverine DOC. Our estimates do not explicitly consider the influence of hydrology and its seasonal variation on the DBC content of riverine DOC. The DBC content of riverine DOC was previously shown to vary across hydrological gradients in one river system; specifically, the Yellow River, where DBC content of DOC varied between 1% in winter and 13% in the high spring flow of 2015^58^. Nonetheless, most studies have failed to identify significant seasonality in the DBC content of DOC, including in major high latitude rivers^54^, tropical rivers and temperate catchments^57,62^. Soil moisture, a proxy for seasonal hydrology, was also not found to be a significant driver of variability in the DBC content of DOC in tropical Brazilian rivers^48^. We verified that the differences in the DBC content of DOC across latitudes and biomes are not due to bias in the timing of sample collection. Such a bias could, in theory, influence the spatial variability that we observe across river classes if one river class disproportionately includes samples from periods with a high DBC content of DOC relative to other river classes. However, we found no evidence for significant variability in the DBC content of DOC across seasons within river classes collated at the global scale (Supplementary

*4*

Figure S3), and hence conclude that seasonality in the DBC content of DOC was insufficient to significantly bias the spatial differences between river classes that we observed in our dataset.

***B) Specific Comments***

1-1

Feb 2, 2020 at 5:04 PM

New finding from prior study. Verify if authors estimate/discuss impact of seasonality of streamflow

on annual estimates of BC fluxes.

**Please see our response provided above (under “Overview”).**

1-2

Significant finding suggesting that once altered by fire, TOC may be exported at a greater rate than

general TOC in watersheds towards the ocean.

*(Response not required)*

1-3

In fact, I would go further and suggest that such processes should be integrated into the modeling of changing conditions due to projected increases in fire dynamics due to climate change. That export component of BC may increase in the future, especially from areas with predicted hire fire incidence and intensity.

**Thank you for this valuable suggestion, which we incorporated in the revised text as follows:**

“Biogeochemical models should be adapted to explicitly represent the unique terrestrial dynamics of black carbon, and these models should be used to quantify the impact of projected changes to regional fire regimes on the input of recalcitrant terrigenous carbon to the global oceans.”

2-1

While this may be true of the refractory, condensed aromatic structures that are predominantly forming BC, not all dissolved pyrogenic OC is derived from microbial degradation of particulate BC in soils. There is a significant fraction of soluble PyC that is released directly from charred organic matter (either in soils or through deposition and dissolution of aerosols). I understand that the authors here discuss the refractory, condensed component of PyC, but mentioning the additional flux of easily soluble PyC can help make a case for an even larger input of total PyC from the landscape to the ocean. The latter flux will be recycled rapidly and potentially contribute to rapid CO2 evasion from rivers and coastal systems.

**We agree that this is worth clarifying the scope of our estimates in the main text, so we added the following paragraph to the section “Global Riverine Export of Black Carbon”:**

“Our estimates account for the organic products of fire whose recalcitrance is considered to differ substantially from that of unburned vegetation (see methods). We do not account for the export of other soluble products of low-temperature vegetation charring, such as levoglucosan, which have short residence times compared to those of the DBC and thus lesser significance for the oceanic storage of terrigenous carbon^40,76,77^.”

3-1

The authors clarify, and estimate, the geographical variability of the DBC flux providing very valuable adjustments to the prior estimates of global DBC fluxes. However, their study does not evaluate the influence of seasonality on DBC fluxes. The assumption that DBC/DOC ratios remains constant over the year and that annual DBC fluxes for any river can be modeled by knowing the DOC fluxes and selected DBC/DOC values is over simplified. This is not a fatal flaw of the study as the authors have demonstrated that the understanding of BC production and cycling in the environment is still evolving.

5

We need such studies to better define the incremental gains in this understanding while at the same time identifying the areas of uncertainty that need constraining. How can the authors estimate the temporal variability in global fluxes?

**Please see our response provided above (under “Overview”).**

5-1

Please clarify that this variability is estimated only for geographical scale. The temporal variability of

these rivers’ fluxes, and thus the global flux, is not estimated or discussed.

**Please see our response provided above (under “Overview”).**

6-1

It would be critical to estimate if those dynamics also change from high to low hydrographs (temporal

scale) in these river systems.

**Please see our response provided above (under “Overview”).**

7-1

The analysis suggest that fires, followed by soil processes leading to the dissolution of BC, have a

disproportionate role in recycling TOC and transferring it to a long-residence time pool in the ocean.

*(Response not required)*

9-1

A very critical statement and contribution to this research and future work. Undoubtedly, the changing

earth climate conditions and fire dynamics will influence DBC fluxes to the soils and ocean.

*(Agreed- and not only climate, but also changes in human factors e.g. deforestation, use of fire in land management – the major challenge to modelling past fire regimes is human rather than climate, feeding into huge uncertainty in future projections.)*

9-2

I urge the authors to evaluate temporal variability (or lack thereof) of DBC/DOC in major rivers to provide the most appropriate parameters for the biogeochemical models that will evaluate the potential future fluxes of DOC and DBC to the ocean. It is understood that this is a recommendation for future work, or to affect the present publication beyond the recognition of the potential influence of temporality (science is incremental and needs to be understood and performed as su such)

**Please see our response provided above (under “Overview”).**

10-1

I fully agree and these processes should include potential temporal variability of hydrographic flow on

DBC/DOC.

**Please see our response provided above (under “Overview”).**

11-1

The low-temperature fraction of the DCB may not respond linearly to DOC hydrographic response (Myers-Pigg et al. 2017). Some discussion needs to address this potential issue. For example, what proportion of the total DBC may occur in the low-T part of the spectrum? How many of the samples

*6*

are taken early, during, late in the peak of the streamflow and which are taken during baseflow periods. What is a potential added source of error due to temporal variability and decoupling of different DCB fractions with DOC?

**Please see our response provided above (under “Overview”).**

**Reviewer #3 (Remarks to the Author):**

The manuscript, from Jones et al., reports estimates of latitudinal- and biome-dependent dissolved black carbon (DBC) exports from rivers into oceans. These estimates were based on meta-analysis of an expanded dataset of 409 observations compiled from published and unpublished data. The authors concluded that both variability across latitude and biome were significant – with rivers in the tropics and high latitude discharging higher amounts of dissolved black carbon to the ocean, than those in temperate latitudes. Though not qualitatively surprising, the authors’ findings provide some semi-quantitative estimates for spatially constraining DBC riverine-ocean flux. There are however, a series of ambiguities that prevents the reviewer from recommending this manuscript for publication without substantial revisions. The major ambiguities are provided below:

1. Beside the reference years, the timing/temporal criteria for a sample to be in the database is not clear. Were the meta-analysis criteria for rivers sampled at a comparable stage (e.g. flood stage)? Were the samples distributed across the year or confined to a given season (e.g. fire or otherwise)? In other words, how does the reviewer ascertain that the spatial (latitudinal and biome) differences reported were not really artefacts of temporally biased sampling arising from incomparable timing (e.g. in dry vs. wet or fire vs. non-fire seasons)?

Thanks very much for this important comment, which was also raised by Reviewer #2. Indeed, it is important to ensure that seasonal bias in sample collection does not influence the spatial patterns that we observe. We have taken the following steps to address this point:

1. We incorporated temporal sampling information (season) into our dataset of DBC and DOC concentration measurements.
2. For studies with multiple sampling periods, we used Tukey HSD tests to identify instances where there was variability across seasons within river classes.
3. We present the results of the above analyses in the supplementary material – please see Supplementary Figure S3.
4. We incorporate the following paragraph into the main text:

“The dataset on which our global export estimates are based captures global-scale spatial variability in the DBC content of riverine DOC. Our estimates do not explicitly consider the influence of hydrology and its seasonal variation on the DBC content of riverine DOC. The DBC content of riverine DOC was previously shown to vary across hydrological gradients in one river system; specifically, the Yellow River, where DBC content of DOC varied between 1% in winter and 13% in the high spring flow of 2015^58^. Nonetheless, most studies have failed to identify significant seasonality in the DBC content of DOC, including in major high latitude rivers^54^, tropical rivers and temperate catchments^57,62^. Soil moisture, a proxy for seasonal hydrology, was also not found to be a significant driver of variability in the DBC content of DOC in tropical Brazilian rivers^48^. We verified that the differences in the DBC content of DOC across latitudes and biomes are not due to bias in the timing of sample collection. Such a bias could, in theory, influence the spatial variability that we observe across river classes if one river class disproportionately includes samples from periods with a high DBC content of DOC relative to other river classes. However, we found no evidence for significant variability in the DBC content of DOC across seasons within river classes collated at the global scale (Supplementary Figure S3), and hence conclude that seasonality in the DBC content of DOC was insufficient to significantly bias the spatial differences between river classes that we observed in our dataset.”

7

2. It is not clear in the main manuscript what 409 observations meant. Are these from 5, 10, 15 rivers? Across 1, 3, 5 years? How do we ascertain that beyond just having a larger n than the incorporated databases, the new “expanded” database has more value? The authors need to clarify and clearly establish why their addition to, and aggregation or disaggregation of, this data to address their questions on DBC flux is valid. Although the authors pointed to the use of non-parametric statistics, such analyses still require standards. It was not clear to the reviewer that these standards were met.

**Re what constitutes an ‘observation’:**

Our dataset includes 409 samples in total:

- 195 of which derive from 34 major rivers and 214 from 151 minor channels
- 405 of which are individual coupled measurements of DOC and DBC and 4 are the average of multiple samples

These samples build on the original dataset of Jaffe et al (upon which we build our dataset), which featured:

- 109 samples in total
- 45 of which derive from 22 major rivers and 64 from 61 minor channels.
- 106 of which are individual coupled measurements of DOC and DBC and 3 average of multiple samples.

We now clarify these features in the final paragraph of the introduction as follows:

“Our dataset incorporates 409 coupled measurements of DOC and DBC concentration in total, including 195 from 34 major rivers and 214 from 44 minor channels (Table S1). The dataset incorporates 300 new coupled measurements from 12 major rivers and 90 minor channels (Table S1). 405 of the data points represent individual samples taken at a single time, while 4 of the data points represent the average of samples collected over multiple seasons (Figure S3). The dataset includes 204 new samples from (sub)tropical rivers (<30° N/S), which is an important advance because ~90% of global BC production occurs in the (sub)tropics^16^ and because (sub)tropical rivers contribute ~60% of the total global DOC export flux (128 ± 20 Tg C year-1 of 205 ± 21 Tg C year-1)^8^.”

We expand supplementary table S1 to also summarise the count of samples for each channel type, and the change versus Jaffe et al. (2013). Information regarding the season of sample collection is provided in the supplementary dataset.

**Re non-parametric statistics:**

We did not use non-parametric statistics to assess the distribution of DBC/DOC% across our dataset. The only information with a non-parametric distribution that was incorporated into our study design derives from the flux estimates of previous studies (as explained in the methods):

- Global BC production by landscape fires is based on the pyrogenic carbon production estimates of Jones et al. (ref. 22; 256!"#

$%& Tg C year^-1^)

- Global POC export flux from Galy et al. (ref. 9; 157!’#

$(& Tg C year^-1^)

- Global PBC export flux from Coppola et al. (ref. 53; 25!)

$*& Tg C year^-1^).

(+ - values here represent the interquartile range of estimates).

In all cases, the non-parametric distributions of these estimates were converted to parametric distributions in the most conservative way possible. The greatest difference between the central and upper and the central and lower estimates were selected to represent uncertainty in both directions.

8

Hence the estimate of Jones et al. becomes 256 ± 84 Tg C year^-1^. The lower bound of the estimated range is conservative.

We also make the assumption that the stated range represents 1σ uncertainty. This is similar to the middle 50% represented by the interquartile range.

All of this information is already available in the methods section, so we chose not to make changes to the text in response to this comment. However, we hope this synthesis/summary helps to clarify our approach.

3. Beyond putting some early global estimates on the long suspected and logical biome-dependent riverine DBC flux; the novelty of the methods and findings are lacking. Even with using a different, more complicated statistical approach to estimate global riverine-ocean DBC flux, the results (in essence) confirmed the previously simpler determined estimates of Jaffe et al.

We strongly disagree with the assessment that “the novelty of the methods and findings are lacking”.

We do indeed show something that is “long-suspected and logical”; however, the word “unproven” should also feature here. Indeed, the fact that the results are suspected, logical, and yet *unproven* to date is one reason why the results will be of such interest to the community.

We expect our novel results to markedly change the understanding of riverine DBC export within the DOC and pyrogenic carbon research communities. Specifically, it advances understanding from a “universal” global relationship stated in Jaffe et al. (2013), often referred to as “coupling”, to identify global-scale spatial variability in the export of DBC as a fraction of DOC. Our study demonstrates large-scale geographical de-coupling of DBC fluxes from those of DOC and constitutes a critical advance to the field. The methods remain relatively simple, yes, however the novelty is not disputable.

These results will inform and direct biogeochemical modelling work, which is the next critical step to understanding the terrestrial dynamics of BC, the export of BC to the global oceans, and how these dynamics differ from bulk OC.

4. The non-DBC sections of the manuscript were at best, hand-waving and really did not add anything to current understanding of BC cycling between terrestrial and oceanic systems. These sections which incidentally made up most of the paper lack a systematic, robust, analytical assessment and more often, than not, felt like a mad dash of grabbing numbers and combining them into a back-of-the-envelope calculation with minimal regard for scientific rigor. This led to a manuscript that started out reasonably well and focused but quickly descended into a frantic quest for plugging “global” gaps outside the scope of the dataset at the expense of developing a robust paper around a single theme.

While it is not clear which sections provoked this comment, it seems most likely to relate to the section “Oceanic Fate of Dissolved Black Carbon”, where we discuss the likelihood of riverine BC persisting in the global oceans following its riverine export.

Here we can appreciate the argument that the discussion is outside of the scope of the *dataset*. We would argue that it is within the scope of the *study*, on the basis that riverine BC export is only relevant from a carbon cycle perspective if the exported BC remains in the oceans for long periods (or, more specifically, periods longer than those of bulk OC).

Nonetheless, we concede that the photochemical loss estimates are ‘back-of-the-envelope’ extrapolations that rely on rates from previous work: our study does not fundamentally advance the understanding of oceanic BC dynamics.

*9*

On balance, we have decided to remove the section “Oceanic Fate of Dissolved Black Carbon” and relevant sections of the methods and supplementary material.

We hope that this satisfies the reviewer by improving the balance of results and discussion in the paper.

5. Figure 1 is beautiful! The reviewer loves it. In the light of authors discussion on BC being more persistent than non-BC, how do they rationalize the rather small fraction of PBC in terrigenous POC?

Thanks!

Oceanic suspended PBC represents ~6% of total suspended POC and ~18% of suspended terrigenous POC, based on global estimates of suspended POC from Honjo et al. (2008), suspended PBC from Coppola et al. (2014) and a terrigenous POC fraction of one-third from Burdige et al. (2007).

For comparison, ocean sediment BC represents ~20% of ocean sediment OC and ~60% of ocean terrigenous sediment OC, based on global estimates of ocean sediment OC from the IPCC/Le Quere et al., 2018, a terrigenous OC fraction of one-third from Burdige et al. (2007), and a best estimate for ocean sediment BC/OC% of 20% (informed by: Coppola et al., 2014, Verardo and Ruddiman, 1996; Masiello and Druffel, 1998, 2003; Gustafsson and Gschwend, 1998; Middelburg et al., 1999; Lohmann et al., 2009).

The simplest explanation for the increase in BC/OC% and BC/OCTerrigenous% would be that suspended PBC is preferentially conserved in the suspended POC pool, relative to both oceanic and non-BC terrigenous OC. - BC would be preferentially deposited and stored in bed sediments.

However, the estimates for terrigenous OC and terrigenous BC stocks are formed independently. This figure is designed to summarise the current state of knowledge of the global BC cycle: studies that quantify both the terrigenous fraction of total OC and the BC fraction of the terrigenous OC are certainly needed. Glad that the figure was thought-provoking.

**Reviewer comments, second version:**

REVIEWERS' COMMENTS:

Reviewer #1 (Remarks to the Author):

I believe the authors have done a satisfactory job of addressing all reviewer comments.


Reviewer #2 (Remarks to the Author):

The authors did a great job at addressing the comments of all reviewers. I am quite impressed by their incorporation of the temporal analysis (which supports their original assumption that temporal variability did not have a significant impact on their interpretation) and their agreement to remove the “Oceanic Fate of Dissolved Black Carbon” section from the discussion. I don’t disagree with Reviewer #3 that this discussion included loose assumptions and broad estimates with a potential for large scale uncertainty. However, I am more of the mind to push science from statements that need to be tested especially when these are shored by strong arguments and data. In my mind, this helps science move forward. But at the same time, I can understand the request for a rigorous and exacting process of science. I applaud the authors for reaching a compromise and focusing on the important message they want to make, the one that is more clearly supported by their data. I encourage them to keep tackling this issue, maybe through the association with modelers to evaluate the impact DBC may have on the global carbon cycle under different scenarios. Well done!

**Author rebuttal, second version:**

**Reviewer #1 (Remarks to the Author):**

I believe the authors have done a satisfactory job of addressing all reviewer comments.


**Reviewer #2 (Remarks to the Author):**

The authors did a great job at addressing the comments of all reviewers. I am quite impressed by their incorporation of the temporal analysis (which supports their original assumption that temporal variability did not have a significant impact on their interpretation) and their agreement to remove the “Oceanic Fate of Dissolved Black Carbon” section from the discussion. I don’t disagree with Reviewer #3 that this discussion included loose assumptions and broad estimates with a potential for large scale uncertainty. However, I am more of the mind to push science from statements that need to be tested especially when these are shored by strong arguments and data. In my mind, this helps science move forward. But at the same time, I can understand the request for a rigorous and exacting process of science. I applaud the authors for reaching a compromise and focusing on the important message they want to make, the one that is more clearly supported by their data. I encourage them to keep tackling this issue, maybe through the association with modelers to evaluate the impact DBC may have on the global carbon cycle under different scenarios. Well done!

We thank the reviewers for their contributions to the review process, and especially for their constructive comments on the first submitted version of our article. Thanks also to reviewer #2 for the kind praise and encouragement expressed above.

**Reviewer comments, third version:**

**Author rebuttal, third version:**
